# Supplementary material for: Adaptation to high rates of chromosomal instability and aneuploidy through multiple pathways in budding yeast
Source: EMBO J. 2022 Dec 19;42(8):e111500. doi: 10.15252/embj.2022111500 (PMC10106982; doi:10.15252/embj.2022111500)
Supplement: Supplementary file 1 — Expanded View Figures PDF [file EMBJ-42-e111500-s004.pdf]

Expanded View Figures

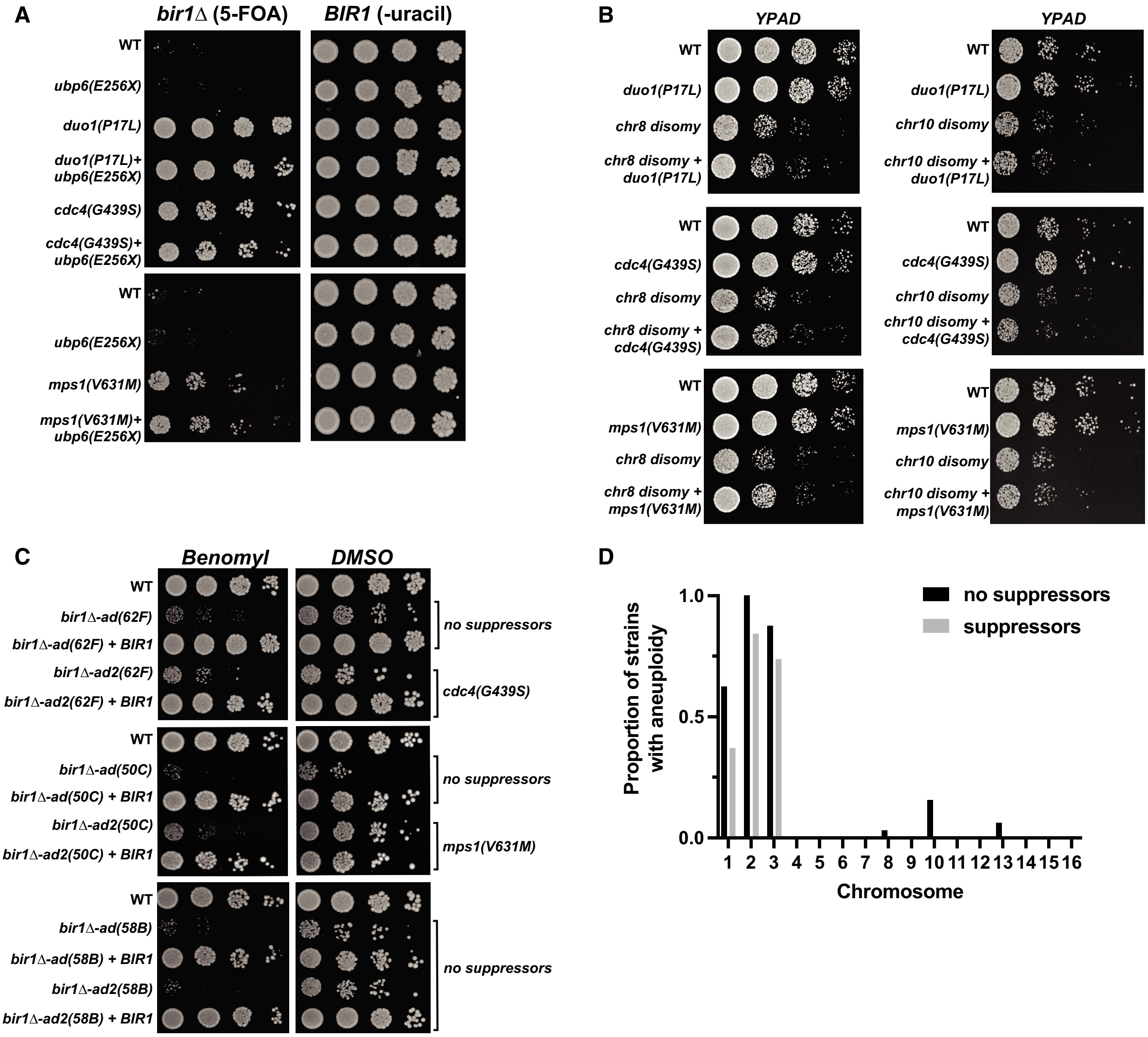

Figure EV1. CIN-related mutations in *bir1Δ-ad2* strains are associated with decreased aneuploidy.

A Ten-fold serial dilutions on the indicated media are shown. Strains engineered with the *ubp6(E256X)* aneuploidy tolerance mutation show no *bir1Δ* suppression. Strains with both the *ubp6(E256X)* mutation and a *bir1Δ* suppressor mutant do not additionally rescue the loss of CPC activity.

B Ten-fold serial dilutions on the indicated media are shown. Strains have disomy of either chromosome 8 (chr8) or chromosome 10 (chr10) with or without the *bir1Δ* suppressor mutations. The combination of either type of disomy with any of the three suppressors shows no change in growth.

C Ten-fold serial dilutions of the indicated strains were made on YPAD plates containing either 0.1% DMSO or 10 μg/ml of benomyl. Strains were adapted to persistent loss of CPC function for different amounts of time with or without *BIR1* then restored. Those *bir1Δ-ad* strains that developed suppressor mutations after the extended growth period (*bir1Δ-ad2(62F)* and *bir1Δ-ad2(50C)*) were better able to cope with benomyl than one that did not develop suppressors (*bir1Δ-ad2*(58B)).

D Histogram showing the proportion of strains that are aneuploid for specific chromosomes in *bir1Δ-ad2* strains that have identified suppressor mutations, and those that do not.

 ©2022 The Authors

Figure EV2. Double mutants of *bir1*Δ suppressors in the Dam1 complex and the *dam1(S20D)* phosphomimic have strong synthetic phenotypes.

A Tetrad dissections performed on diploids with both *sli15*Δ and the *duo1(P17L)* did not produce any viable spores with either *sli15*Δ or a combination of *sli15*Δ and *duo1(P17L)*.

B Cell cycle progression as measured by percentage of large budded cells over time after release from G1 arrest. None of the tested suppressor mutants cause a mitotic delay.

C Tetrad dissections of diploids heterozygous for both a *bir1*Δ suppressor mutant and the *dam1(S20D)* phosphomimic mutant. Presence of either the suppressor mutant or *dam1(S20D)* was determined by antibiotic resistance conferred by a gene integrated downstream of the mutations (clonNAT and G418 respectively). Colonies from haploid spores containing either *dad2(K11Q)*, *dam1(S20D)* or *duo1(P17L)*, *dam1(S20D)* were extremely rare. Spore viability of each genotype was counted in tetrads whose type (parental ditype, nonparental ditype, or tetratype) could be distinguished. Quantification for each mutant is from a single plate, and the WT numbers are the averages from all six plates. The representative image to the right of the graph depicts six tetrads, demonstrating examples of three tetratypes, two nonparental ditypes, and a single parental ditype. In this example, the expectant *duo1(P17L)*, *dam1(S20D)* double-mutant formed a colony from 0 out of seven spores.

D Proportion of colonies that grow on plates with restrictive (lacking uracil) versus permissive (YPAD) media after 24 h growth under permissive conditions with a *URA3*-containing plasmid. *duo1(P17L)*, *dam1(S20D)* double mutants have a strong decrease in transmission fidelity. Data are from three independent experiments Means and standard deviations are shown.

E Cell cycle progression as measured by percentage of large budded cells over time after release from G1 arrest. Rare surviving *duo1(P17L)*, *dam1(S20D)* double mutants have a strong mitotic delay. Wild-type (WT) cells treated with nocodazole and benomyl were used as a positive control for mitotic arrest.

F Serial dilution showing growth of strains containing either the double-mutant (*duo1(P17L)*, *dam1(S20D)*), the *ipl1-321* temperature-sensitive allele, or a combination of all three mutations. At the restrictive temperature (34°C), the double-mutant rescues growth of the *ipl1-321* allele. Ten-fold serial dilutions were done on YPAD plates at the indicated temperatures and were grown for 48 h.

Data information: (ns) not significant; (*) $P < 0.05$; (**) $P < 0.01$; (***) $P < 0.001$; unpaired t-test.

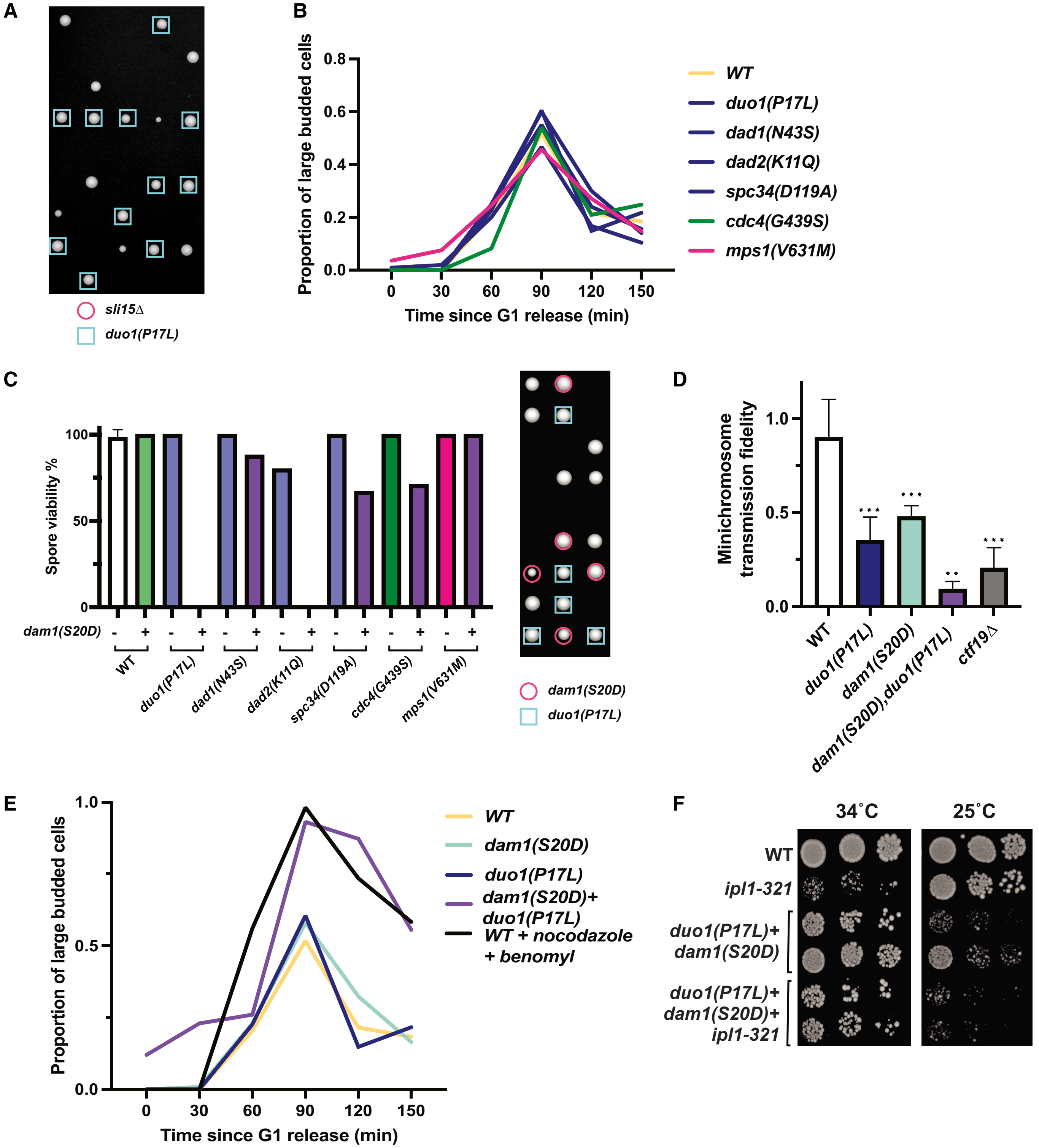

Figure EV2.

 ©2022 The Authors

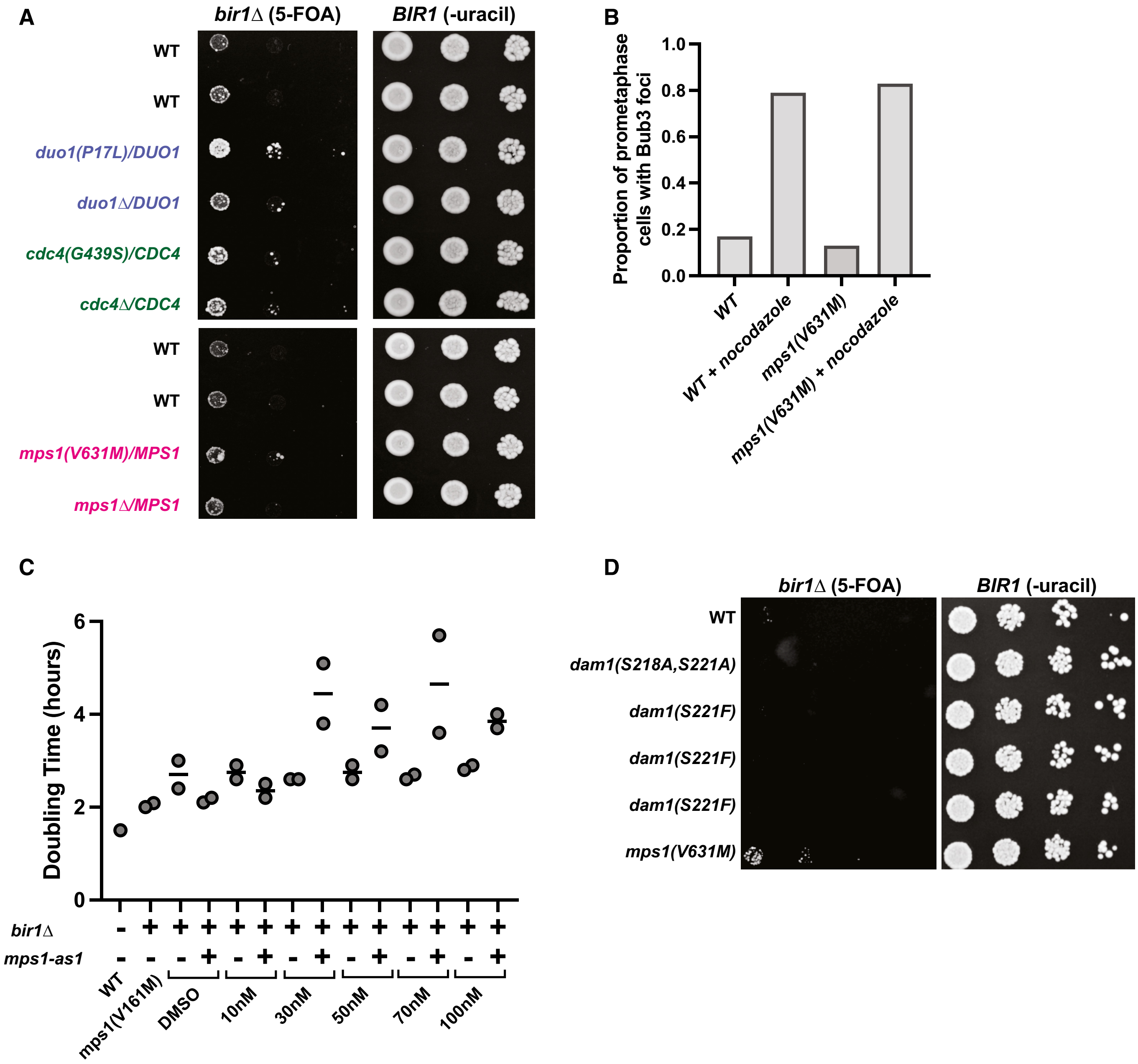

Figure EV3. Suppression of *bir1Δ* by Mps1 mutations occurs through decreased kinase activity that does not inhibit spindle assembly checkpoint activity.

A Ten-fold serial dilutions on the indicated media are shown. Strains engineered with the indicated mutations were tested for rescue of *BIR1* deletion. Diploid strains were heterozygous for either an identified suppressor mutation or a full deletion of the gene.

B Quantification of the proportion of prometaphase cells with Bub3-mNeonGreen foci with or without 15 μg/ml nocodazole. The *mps1(V631M)* mutation does not affect the percentage of cells with spindle assembly checkpoint foci. Data are from a single experiment.

C Growth assays conducted with an ATP-analog (1NM-PP1) that specifically inhibits the *mps1-as1* allele. Strains with wild-type Mps1 were not affected by the inhibitor. The WT strain is CCY1905. Data are from two independent experiments.

D Ten-fold serial dilutions on the indicated media are shown. Strains engineered with the indicated mutations were tested for rescue of *BIR1* deletion. Dam1 alleles with nonphosphorylatable mutations in Mps1 phosphosites (S218 and S221) do not rescue growth after *bir1Δ*.

©2022 The Authors

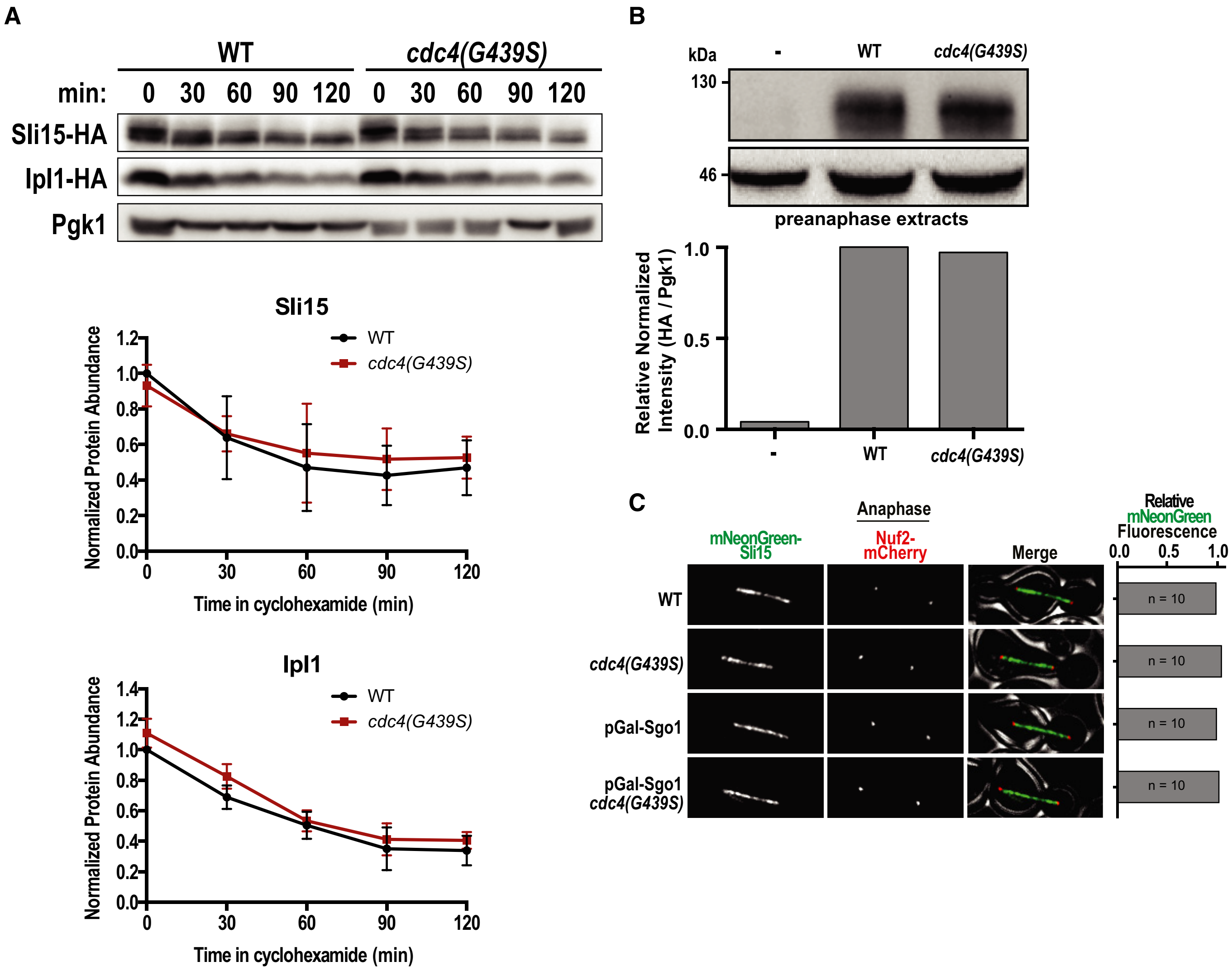

Figure EV4. *bir1Δ* suppressor mutations in Cdc4 do not affect protein stability of Sli15 or Ipl1.

A Western-blot of a time series of Ipl1 and Sli15 protein levels after inhibition of protein translation with cycloheximide. Cycloheximide was added to cells at time point 0, and cells were harvested and fixed at 30 min intervals. Representative images from one experiment are shown on top and quantification of three independent experiments is shown on bottom. Error bars represent standard deviation of three biological replicates from Western Blot quantifications.

B Western-blot and quantification of mNeonGreen-HA-Sli15 expression levels in *cdc4(G439S)* expressing cells. Pre-anaphase protein extracts were prepared as in Fig 6B and probed for Sli15 expression levels via immunoblotting using an HA antibody. Data are from a single experiment.

C The amount of mNeonGreen-Sli15 spindle localization was measured in anaphase. Sgo1 was depleted by placing the gene under a galactose-inducible promoter and switching to glucose-containing media (YPAD). For quantifications, line-scans perpendicular to the spindle were measured. Representative images are on the left. Scale bar is 2 μm. Data are from a single experiment.

Figure EV5. Engineering and characterization of FBXW7 mutant cell lines.

A Strategy implemented to introduce specific point mutations in *FBXW7* in HAP1 cells. Transfected cell lines were originally screened for a silent mutation that creates an EcoR1 restriction site.

B Sanger sequencing confirms the presence of the G437S mutation in *FBXW7*.

C Cell lines mutant for *FBXW7* have an increased colony size after 12 days of growth in comparison to a cell line with wild-type *FBXW7*. Means and standard deviations are shown. Each individual value point is the size measurement of a single clone. Data are from two independent experiments. (***) $P < 0.001$; (****) $P < 0.0001$; unpaired *t*-test.

©2022 The Authors

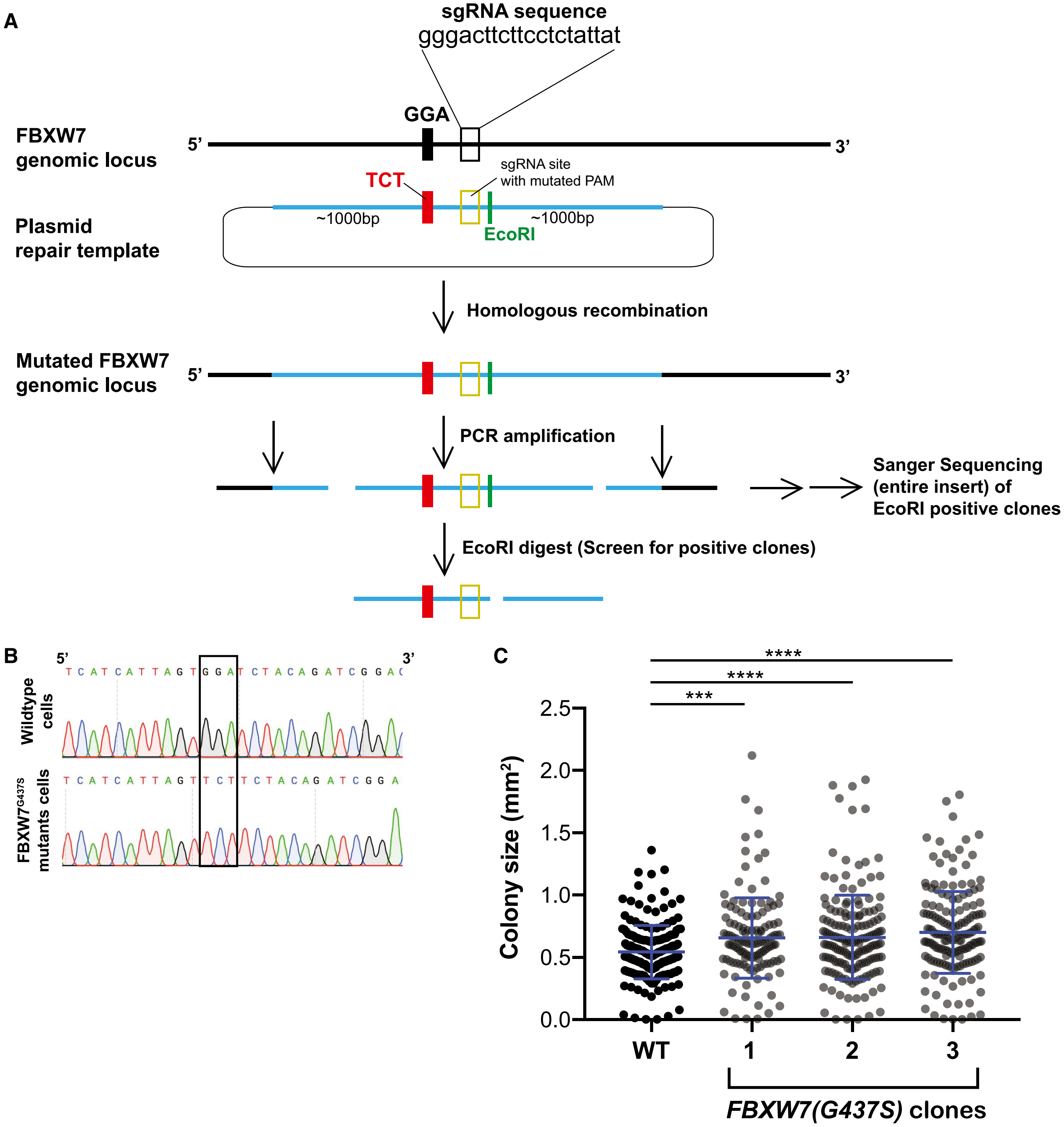
A
sgRNA sequence
gggacttcttcctctattat
GGA
FBXW7
genomic locus
5'
3'
TCT
sgRNA site
with mutated PAM
Plasmid
repair template
~1000bp
~1000bp
EcoRI
Homologous recombination
Mutated FBXW7
genomic locus
PCR amplification
Sanger Sequencing
(entire insert) of
EcoRI positive clones
EcoRI digest (Screen for positive clones)
B
Wildtype
cells
FBXW7G437S
mutants cells
C
Colony size (mm2)
WT
1
2
3
FBXW7(G437S) clones
***
****
****

Figure EV5.
