# Supplementary material for: Adaptation to high rates of chromosomal instability and aneuploidy through multiple pathways in budding yeast
Source: EMBO J. 2022 Dec 19;42(8):e111500. doi: 10.15252/embj.2022111500 (PMC10106982; doi:10.15252/embj.2022111500)
Supplement: Supplementary file 2 — Table EV1 [file EMBJ-42-e111500-s001.docx]

**Table EV1. Yeast strains, Cell lines and Plasmids from this study**

| **Strain** | **Genotype** | **Source** | **Background** | **Figure/s** |
| --- | --- | --- | --- | --- |
| CCY149 | MATa/α; Bir1+/Δ::HYG; ura3-1; LEU2/leu2,3-112; his3-11:pCUP1-GFP12-lacI12:HIS3 trp1-1:256lacO:TRP1; LYS2/lys2Δ ADE2/ade2-1; can1-100; bar1Δ | a | W303 | 1; 2; EV1 |
| *bir1∆* haploids | These strains were made either through tetrad dissection of CCY149 or from single colonies of CCY1905 selected on FOA plates. | c | W303 | 1B;1C;1D |
| *bir1∆-ad* haploids | All strains were derived from tetrad dissecting CCY149 (102 haploid strains) | b | W303 | 1B;1C;1D;1E;1F;1G;2F; EV1C |
| *bir1∆-ad2* haploids | All strains were derived from bir1∆-ad (68 strains) | b | W303 | 1B;1C;1D;1E;1F;1G;2D;2E;2F;EV1C; EV1D |
| *bir1∆-ad3* haploids | All strains were derived from bir1∆-ad2 (15 strains) | c | W303 | 1B;1C;1D;1E;1F;1G;2D;2E;2F EV1C; EV1D |
| CCY434 | MATa; ura3-1; leu2;3-112; his3-11; trp1-1; ade2-1 | a | W303 | 3A; 4D; EV2B; EV2E; EV2F |
| CCY747 | MATα; his3-11:pCUP1-GFP12-lacI12:HIS3 trp1-1:256lacO:TRP1; leu2,3-112; lys2Δ; ura3-1; ADE2; trp1-1 | a | W303 | 1C;1D;2D;2E; EV1C |
| CCY1744 | MATa/α; Bir1Δ/Δ::G418/HYG; ura3-1; LEU2/leu2,3-112; his3-11:pCUP1-GFP12-lacI12:HIS3 trp1-1:256lacO:TRP1 LYS2/lys2Δ; ADE2/ade2-1; can1-100; bar1Δ; pCC598::URA3 | a | W303 | EV3A |
| CCY1887 | MATa; ura3-1; leu2,3-112; trp1-1; ade2-1; pGalCEN8::URA3::HIS3(pCC631) | b | W303 | EV1B |
| CCY1905 | MATa; Bir1Δ:HYG; ura3-1; leu2,3-112: pCUP1-GFP12-lacI12:HIS3 trp1-1:256lacO: lys2Δ; can1-100 bar1Δ; pCC598::URA3 | b | W303 | 2B; 4B; 6A; EV1A; EV3C; EV3D |
| CCY1934 | CCY1905 + UBP6::ubp6(E256X):G418 | c | W303 | EV1A |
| CCY1946 | Bir1Δ:HYG; ura3-1; leu2,3-112: pCUP1-GFP12-lacI12:HIS3 trp1-1:256lacO: lys2Δ; can1-100 bar1Δ; pCC598::URA3; pGalCEN10::LYS2::pCC644::LEU2 | b | W303 | EV1B |
| CCY2999 | MATa Bir1Δ:HYG ; ura3-1; leu2;3-112: pCUP1-GFP12-lacI12:HIS3 trp1-1:256lacO:TRP1 lys2Δ ADE2 can1-100 bar1Δ | c | W303 | EV3C |
| CCY3001 | CCY2999 + mps1-as1:G418 | c | W303 | EV3C |
| CCY3031 | CCY1905 + duo1(P17L):G418 | c | W303 | 2B; 4B; EV1A |
| CCY3033 | CCY1905 + cdc34(M64T):G418 | c | W303 | 2B |
| CCY3035 | CCY1905 + mif2(D241I):G418 | c | W303 | 2B |
| CCY3037 | CCY1905 + ndc80(K181N):G418 | c | W303 | 2B |
| CCY3039 | CCY1905 + spc105(R583G):G418 | c | W303 | 2B |
| CCY3041 | CCY1905 + sli15(G334S):G418 | c | W303 | 2B |
| CCY3043 | CCY1905 + sli15(L71S):G418 | c | W303 | 2B |
| CCY3047 | CCY1905 + cdc4(G439S):G418 | c | W303 | 2B; 6A; EV1A |
| CCY3048 | CCY1905 + cdc4(S438G):G418 | c | W303 | 2B |
| CCY3050 | CCY1905 + mps1(R596H):G418 | c | W303 | 2B |
| CCY3052 | CCY1905 + mps1(V631M):G418 | c | W303 | 2B; EV1A; EV3C; EV3D |
| CCY3064 | CCY1905 + spc34(D119A):G418 | c | W303 | 2B |
| CCY3083 | CCY1905 + rtg2(A433P):G418 | c | W303 | 2B |
| CCY3085 | CCY1905 + spc97(S816X):G418 | c | W303 | 2B |
| CCY3210 | MATa/α; Bir1Δ/Δ:HYG ; ura3-1/ura3-1; leu2;3-112: pCUP1-GFP12-lacI12:HIS3/leu2;3-112 trp1-1:256lacO:TRP1/trp1-1:256lacO: lys2Δ ade2-1/ADE2 can1-100/can1-100 bar1Δ/bar1Δ; pCC598::URA3 DUO1/duo1(P17L):G418 | c | W303 | EV3A |
| CCY3211 | MATa/α; Bir1Δ/Δ:HYG ; ura3-1/ura3-1; leu2;3-112: pCUP1-GFP12-lacI12:HIS3/leu2;3-112 trp1-1:256lacO:TRP1/trp1-1:256lacO: lys2Δ ade2-1/ADE2 can1-100/can1-100 bar1Δ/bar1Δ; pCC598::URA3 CDC4/cdc4(G439S):G418 | c | W303 | EV3A |
| CCY3212 | MATa/α; Bir1Δ/Δ:HYG ; ura3-1/ura3-1; leu2;3-112: pCUP1-GFP12-lacI12:HIS3/leu2;3-112 trp1-1:256lacO:TRP1/trp1-1:256lacO: lys2Δ ade2-1/ADE2 can1-100/can1-100 bar1Δ/bar1Δ; pCC598::URA3 MPS1/mps1(V631M):G418 | c | W303 | EV3A |
| CCY3299 | MATa; ura3-1; leu2;3-112; his3-11; trp1-1; ade2-1; lys2; ipl1-321; duo1(P17L):G418 | c | W303 | 3A |
| CCY3356 | MATa; ura3-1; leu2;3-112; his3-11; trp1-1; ade2-1; lys2; ipl1-321; cdc4(G439S):G418 | c | W303 | 3A |
| CCY3358 | MATa; ura3-1; leu2;3-112; his3-11; trp1-1; ade2-1; lys2; ipl1-321; mps1(V631M):G418 | c | W303 | 3A |
| CCY3439 | MATa/α; Bir1Δ/Δ:HYG ; ura3-1/ura3-1; leu2;3-112: pCUP1-GFP12-lacI12:HIS3/leu2;3-112 trp1-1:256lacO:TRP1/trp1-1:256lacO: lys2Δ ade2-1/ADE2 can1-100/can1-100 bar1Δ/bar1Δ; pCC598::URA3 DUO1/duo1∆::NAT | c | W303 | EV3A |
| CCY3441 | MATa/α; Bir1Δ/Δ:HYG ; ura3-1/ura3-1; leu2;3-112: pCUP1-GFP12-lacI12:HIS3/leu2;3-112 trp1-1:256lacO:TRP1/trp1-1:256lacO: lys2Δ ade2-1/ADE2 can1-100/can1-100 bar1Δ/bar1Δ; pCC598::URA3 CDC4/cdc4∆::NAT | c | W303 | EV3A |
| CCY3443 | MATa/α; Bir1Δ/Δ:HYG ; ura3-1/ura3-1; leu2;3-112: pCUP1-GFP12-lacI12:HIS3/leu2;3-112 trp1-1:256lacO:TRP1/trp1-1:256lacO: lys2Δ ade2-1/ADE2 can1-100/can1-100 bar1Δ/bar1Δ; pCC598::URA3 MPS1/mps1::NAT | c | W303 | EV3A |
| CCY3445 | MATα; ura3-1; leu2;3-112; his3-11; trp1-1; ade2-1; ura3-1; leu2;3-112; his3-11; trp1-1; ade2-1; LYS2+; BUB3-mNeonGreen:NAT; NUF2-mRuby3:Hygro | c | W303 | 3B; 4C; EV3B |
| CCY3469 | CCY1905 + dam1-765(S221F):G418 | c | W303 | EV3D |
| CCY3643 | CCY3445 + duo1(P17L):G418 | c | W303 | 3B; 4C |
| CCY3644 | CCY3445 + cdc4(G439S):G418 | c | W303 | 3B |
| CCY3645 | CCY3445 + mps1(V631M):G418 | c | W303 | 3B; EV3B |
| CCY3646 | CCY3445 + dam1(3D):G418 | c | W303 | 4C |
| CCY3684 | MATa; ura3-1; leu2;3-112; his3-11; trp1-1; ade2-1; cdc4(G439S):G418 | c | W303 | EV2B |
| CCY3688 | CCY1905 + dad1(N43S):G418 | c | W303 | 2B |
| CCY3694 | CCY1905 + ask1(S216F):G418 | c | W303 | 2B |
| CCY3730 | MATa; ura3-1; leu2;3-112; his3-11; trp1-1; ade2-1; LYS2+; mps1(V631M):G418 | c | W303 | EV2B |
| CCY3736 | MATa; ura3-1; leu2;3-112; his3-11; trp1-1; ade2-1; LYS2+; dam1(3D):G418 | c | W303 | 4D |
| CCY3745 | MATa; ura3-1; leu2;3-112; his3-11; trp1-1; ade2-; LYS2+; duo1(P17L):G418 | c | W303 | 4D; EV2B; EV2E |
| CCY3750 | CCY1905 + dad2(K11Q):G418 | c | W303 | 2B |
| CCY3808 | MATα; ura3-1; leu2;3-112; his3-11; trp1-1; ade2-1; LYS2+; NUF2-mRuby3:Hygro; DAD3-mNeonGreen:NAT | c | W303 | 5B; 5C; 5D |
| CCY3809 | CCY3808 + duo1(P17L):G418 | c | W303 | 5B; 5C; 5D |
| CCY3813 | CCY3808 + cdc4(G439S):G418 | c | W303 | 5B; 5C; 5D |
| CCY3816 | CCY3808 + mps1(V631M):G418 | c | W303 | 5B; 5C; 5D |
| CCY3821 | CCY3808 + dad1(N43S):G418 | c | W303 | 5B; 5C; 5D |
| CCY3852 | MATa; ura3-1;leu2;3-112;his3-11;trp1-1;ade2-1;ADE2;Nuf2-mCherry::G418;pGal-3HA-Sli15::HIS3 | c | W303 | EV4B |
| CCY3880 | MATa; ura3-1; leu2;3-112; his3-11; trp1-1; ade2-1; lys2Δ; ipl1-321 | c | W303 | 3A; EV2F |
| CCY3910 | CCY3445 + dam1(S20D):G418 | c | W303 | 4C |
| CCY3917 | CCY1905 + dam1(S218A;S221A):G418 | c | W303 | EV3D |
| CCY3921 | MATa; ura3-1; his3-11, ade2-1; lys2Δ; can1-100 bar1Δ; ipl1-321; PDS1-18myc::LEU2; trp1-1:lacO:TRP1; dad2(K11Q):G418 | c | W303 | 3A |
| CCY3950 | MATa; ura3-1; leu2;3-112; trp1-1:256lacO: lys2Δ; ade2-1; can1-100 bar1Δ; pCUP1-GFP12-lacI12:HIS3; ipl1-321; dad1(N43S):G418; | c | W303 | 3A |
| CCY4093 | MATa, ura3-1; leu2;3-112; his3-11; trp1-1; dam1(S20D):G418 | c | W303 | 4D; EV2E |
| CCY4106 | MATa; ipl1-321; PDS1-18myc::LEU2; trp1-1:lacO:TRP1; ura3-1; leu2;3-112: pCUP1-GFP12-lacI12:HIS3 trp1-1:256lacO: lys2Δ; spc34(D119A):G418 | c | W303 | 3A |
| CCY4108 | CCY3808 + dad2(K11Q):G418; | c | W303 | 5B; 5C; 5D |
| CCY4201 | MATα; ura3-1; leu2;3-112; his3-11; trp1-1; ade2-1; Sli15-6HA::NAT; Ipl1-3HA::HYG | c | W303 | EV4A |
| CCY4254 | CCY3808 + dam1(S20D):G418 | c | W303 | 5B; 5C; 5D |
| CCY4271 | MATa PDS1-18myc::LEU2; ade2-1; his3-11; dam1(S20D):G418; DAD3-mNeon:NAT; duo1(P17L):G418 | c | W303 | EV2E; EV2F |
| CCY4329 | MATa; ura3-1; leu2;3-112; his3-11; trp1-1; ade2-1; Sli15-6HA::NAT; Ipl1-3HA::HYG; cdc4(G439S):G418; | c | W303 | EV4A |
| CCY4834 | MATa; ura3-1; leu2;3-112; his3-11; trp1-1; ade2-1; dad1(N43S):G418 | c | W303 | EV2B |
| CCY4854 | MATα; ura3-1; leu2;3-112; his3-11; trp1-1; ade2-1; BUB3-mNeonGreen:NAT; NUF2-mRuby3:Hygro; dad1(N43S):G418 | c | W303 | 3B |
| CCY4863 | MATα; ura3-1; leu2;3-112; his3-11; trp1-1; ade2-1; BUB3-mNeonGreen:NAT; NUF2-mRuby3:Hygro; spc34(D119A):G418 | c | W303 | 3B |
| CCY4911 | MATa; ura3-1; leu2;3-112; his3-11; trp1-1; ade2-1; pRS316::URA3 | c | W303 | 2C; 3C; 4E; EV2D |
| CCY4913 | CCY4911 + duo1(P17L):G418 | c | W303 | 3C; 4E; EV2D |
| CCY4915 | CCY4911 + dad1(N43S):G418 | c | W303 | 3C |
| CCY4921 | CCY4911 + cdc4(G439S):G418 | c | W303 | 3C |
| CCY4923 | CCY4911 + mps1(V631M):G418 | c | W303 | 3C |
| CCY4925 | CCY4911 + dam1(S20D):G418 | c | W303 | 4E; EV2D |
| CCY4927 | CCY4911 + dam1(3D):G418 | c | W303 | 4E |
| CCY4929 | MATa; PDS1-18myc::LEU2; DAM1::dam1(S20D):G418; DAD3-mNeonGreen:NAT; DUO1::duo1(P17L):G418; pRS316::URA3 | c | W303 | EV2D |
| CCY4933 | CCY4968 + duo1(P17L):G418 | c | W303 | 2C |
| CCY4935 | CCY4968 + dad1(N43S):G418 | c | W303 | 2C |
| CCY4937 | CCY4968 + dad2(K11Q):G418 | c | W303 | 2C |
| CCY4939 | CCY4968 + spc34(D119A):G418 | c | W303 | 2C |
| CCY4947 | CCY4968 + mps1(V631M):G418 | c | W303 | 2C |
| CCY4949 | CCY4968 + cdc4(G439S):G418 | c | W303 | 2C |
| CCY4968 | MATa; Bir1∆:HYG; ura3-1; leu2;3-112: pCUP1-GFP12-lacI12:HIS3 trp1-1:256lacO: lys2∆; can1-100 bar1∆; pRS316::URA3 | c | W303 | 2C |
| CCY4997 | MATa; Bir1∆:HYG; ura3-1; leu2;3-112: pCUP1-GFP12-lacI12:HIS3; can1-100; bar1∆; pCC598::URA3; leu2;3-112; trp1-1:lacO:TRP1; lys2∆; dam1(S20D):G418 | c | W303 | 4B |
| CCY5001 | MATa; Bir1∆:HYG; ura3-1; leu2;3-112: pCUP1-GFP12-lacI12:HIS3; trp1-1; pCC598::URA3; dam1(3D):G418 | c | W303 | 4B |
| CCY5101 | MATa/α; ura3-1; leu2;3-112; his3-11; trp1-1; ade2-1; ura3-1; leu2;3-112; his3-11; trp1-1; DAM1/dam1(S20D):G418; DUO1/duo1(P17L):NAT | c | W303 | EV2C |
| CCY5107 | MATa/α; ura3-1; leu2;3-112; his3-11; trp1-1; ade2-1; ura3-1; leu2;3-112; his3-11; trp1-1; DAM1/dam1(S20D):G418; CDC4/cdc4(G439S):NAT | c | W303 | EV2C |
| CCY5109 | MATa/α; ura3-1; leu2;3-112; his3-11; trp1-1; ade2-1; ura3-1; leu2;3-112; his3-11; trp1-1; DAM1/dam1(S20D):G418; MPS1/mps1(V631M):NAT | c | W303 | EV2C |
| CCY5119 | MATa/α; ura3-1; leu2;3-112; his3-11; trp1-1; ade2-1; ura3-1; leu2;3-112; his3-11; trp1-1; DAM1/dam1(S20D):G418; DAD1/dad1(N43S):NAT | c | W303 | EV2C |
| CCY5282 | MATa; ura3-1; leu2;3-112; his3-11; trp1-1; ade2-1; pRS316::URA3; ctf19∆:G418 | c | W303 | 2C; 3C; 4E; EV2D |
| CCY5326 | MATa; ura3-1; leu2;3-112; his3-11; trp1-1; ade2-1; ADE2; Nuf2-mCherry::G418; pGal1-3HA-Sli15::HIS3; mNeonGreen-6HA-Sli15::URA3; pGal-Sgo1::HYG | c | W303 | 6B; EV4C |
| CCY5373 | CCY3445 + dad2(K11Q):G418 | c | W303 | 3B |
| CCY5376 | MATa; ura3-1; leu2;3-112; his3-11; trp1-1; ade2-1; spc34(D119A):G418 | c | W303 | EV2B |
| CCY5377 | MATa; ura3-1; leu2;3-112; his3-11; trp1-1; ade2-1; dad2(K11Q):G418 | c | W303 | EV2B |
| CCY5420 | MATa; ura3-1; leu2;3-112; his3-11; trp1-1; ade2-1; spc34(D119A):G418; pRS316::URA3 | c | W303 | 3C |
| CCY5422 | MATa; ura3-1; leu2;3-112; his3-11; trp1-1; ade2-1; dad2(K11Q):G418; pRS316::URA3 | c | W303 | 3C |
| CCY5424 | MATa/α; Bir1Δ/Bir1Δ:HYG; ura3-1; leu2,3-112: pCUP1-GFP12-lacI12:HIS3 trp1-1:256lacO: lys2Δ can1-100 bar1Δ; pCC598::URA3 | c | W303 | EV3A |
| CCY5435 | MATa; ura3-1; leu2;3-112; his3-11; trp1-1; ade2-1; ADE2; Nuf2-mCherry::G418; pGal1-3HA-Sli15::HIS3; mNeonGreen-6HA-Sli15::URA3 | c | W303 | 6B; EV4B; EV4C |
| CCY5469 | MATa; ura3-1; leu2;3-112; his3-11; trp1-1; ade2-1; ADE2; Nuf2-mCherry::G418; pGal1-3HA-Sli15::HIS3; mNeonGreen-6HA-Sli15::URA3; pGal-Sgo1::HYG cdc4(G439S):NAT | c | W303 | 6B; EV4C |
| CCY5508 | MATa; ura3-1; leu2;3-112; his3-11; trp1-1; ade2-1; ADE2; Nuf2-mCherry::G418; pGal1-3HA-Sli15::HIS3; mNeonGreen-6HA-Sli15::URA3; cdc4(G439S):NAT | c | W303 | 6B;EV4B; EV4C |
| CCY5512 | MATa/α; ura3-1; leu2;3-112; his3-11; trp1-1; ade2-1; ura3-1; leu2;3-112; his3-11; trp1-1; dad2(K11Q):NAT; dam1(S20D):G418 | c | W303 | EV2C |
| CCY5518 | MATa/α; ura3-1; leu2;3-112; his3-11; trp1-1; ade2-1; ura3-1; leu2;3-112; his3-11; trp1-1; spc34(D119A):NAT; dam1(S20D):G418 | c | W303 | EV2C |
| CCY5524 | CCY3808 + spc34(D119A):G418 | c | W303 | 5B; 5C; 5D |
| CCY5532 | CCY1905 + met30-6:G418 | c | W303 | 6A |
| CCY5535 | CCY1905 + cdc4-1:G418 | c | W303 | 6A |
| CCY5678 | CCY1887 + duo1(P17L):NAT | c | W303 | EV1B |
| CCY5680 | CCY1887 + cdc4(G439S)NAT | c | W303 | EV1B |
| CCY5682 | CCY1887 + mps1(V631M):NAT | c | W303 | EV1B |
| CCY5684 | CCY1946 + duo1(P17L):NAT | c | W303 | EV1B |
| CCY5686 | CCY1946 + cdc4(G439S):NAT | c | W303 | EV1B |
| CCY5688 | CCY1946 + mps1(V631M):NAT | c | W303 | EV1B |
| CCY5690 | bir1∆-ad(50C) + pCC598::URA3 | c | W303 | EV1C |
| CCY5692 | bir1∆-ad2(50C) + pCC598::URA3 | c | W303 | EV1C |
| CCY5694 | bir1∆-ad(62F) + pCC598::URA3 | c | W303 | EV1C |
| CCY5696 | bir1∆-ad2(62F) + pCC598::URA3 | c | W303 | EV1C |
| CCY5698 | bir1∆-ad(58B) + pCC598::URA3 | c | W303 | EV1C |
| CCY5700 | bir1∆-ad2(58B) + pCC598::URA3 | c | W303 | EV1C |
| CCY5706 | MATa; Bir1Δ:HYG; ura3-1; leu2,3-112: pCUP1-GFP12-lacI12:HIS3 trp1-1:256lacO: lys2Δ; can1-100 bar1Δ; pCC598::URA3; DUO1::duo1(P17L):NAT; UBP6::ubp6(E256X):G418 | c | W303 | EV1A |
| CCY5708 | MATa; Bir1Δ:HYG; ura3-1; leu2,3-112: pCUP1-GFP12-lacI12:HIS3 trp1-1:256lacO: lys2Δ; can1-100 bar1Δ; pCC598::URA3; CDC4::cdc4(G439S):NAT; UBP6::ubp6(E256X):G418 | c | W303 | EV1A |
| CCY5710 | MATa; Bir1Δ:HYG; ura3-1; leu2,3-112: pCUP1-GFP12-lacI12:HIS3 trp1-1:256lacO: lys2Δ; can1-100 bar1Δ; pCC598::URA3; MPS1::mps1(V631M):NAT; UBP6::ubp6(E256X):G418 | c | W303 | EV1A |
| CCY5713 | MATa/α; LEU2/ley2,3-112;his3-11:pCUP-GFP12-lacI:HIS3; trp1-1:256lacO:TRP1; lys2∆; ADE2/ade2-1;can1-100 bar1D; BUB1::HYG; DUO1::duo1(P17L):G418; SLI15::NAT | c | W303 | EV2A |
| CCY5736 | CCY5678 + chr8 disomy | c | W303 | EV1B |
| CCY5738 | CCY5680 + chr8 disomy | c | W303 | EV1B |
| CCY5740 | CCY5682 + chr8 disomy | c | W303 | EV1B |
| CCY5742 | CCY5684 + chr10 disomy | c | W303 | EV1B |
| CCY5744 | CCY5686 + chr10 disomy | c | W303 | EV1B |
| CCY5746 | CCY5688 + chr10 disomy | c | W303 | EV1B |
| CCY5752 | MATα; ura3-1; leu2;3-112; his3-11; trp1-1; ade2-1; LYS2+; DUO1::duo1(P17L):NAT; ipl1-321;DAM1::dam1(S20D):G418 | c | W303 | EV2F |
| CCY5754 | MATα; ura3-1; leu2;3-112; his3-11; trp1-1; ade2-1; LYS2+; DUO1::duo1(P17L):NAT; ipl1-321;DAM1::dam1(S20D):G418 | c | W303 | EV2F |
| CCY5755 | MATα; ura3-1; leu2;3-112; his3-11; trp1-1; ade2-1; LYS2+;DUO1::duo1(P17L):NAT;DAM1::dam1(S20D):G418 | c | W303 | EV2F |
| CCY5756 | CCY1887 + chr8 disomy | c | W303 | EV1B |
| CCY5759 | CCY1946 + chr10 disomy | c | W303 | EV1B |

| **Cell line** | **Description** | **Source** | **Figure/s** |
| --- | --- | --- | --- |
| Clone C3 | HAP1 TP53^-^ | e | 6C; EV5C |
| CCH2248 | HAP1 TP53^-^, FBXW7^G437S^(clone 1) | c | 6C; EV5C |
| CCH2251 | HAP1 TP53^-^, FBXW7^G437S^(clone 2) | c | 6C; EV5C |
| CCH2254 | HAP1 TP53^-^, FBXW7^G437S^(clone 3) | c | 6C; EV5C |

| **Plasmid** | **Description** | **Source** |
| --- | --- | --- |
| pRS306 | pBluescript + URA3 | d |
| pRS316 | pBluescript + URA3 | d |
| pCC598 | BIR1 + 1000 bases upstream in pRS306 | b |
| pCC-H046 | Repair plasmid for FBXW7^G437S^ | c |

a – Campbell and Desai; Nature 497: 118-121 (2013)

b – Ravichandran et al; Genes and Development (2018)

c – This study

d – Sinorski and Hieter Genetics 122: 19-27 (1989)

e – J. Loizou lab
