# Supplementary material for: Adaptation to high rates of chromosomal instability and aneuploidy through multiple pathways in budding yeast
Source: EMBO J. 2022 Dec 19;42(8):e111500. doi: 10.15252/embj.2022111500 (PMC10106982; doi:10.15252/embj.2022111500)
Supplement: Supplementary file 6 [file EMBJ-42-e111500-s006.pdf]

THE EMBO JOURNAL

Adaptation to high rates of chromosomal instability and aneuploidy through multiple pathways in budding yeast

Matthew Clarke, Theodor Marsoner, Manuel Alonso Y Adell, Madhwesh Ravichandran, and Christopher Campbell
DOI: 10.15252/embj.2022111500

Corresponding author(s): Christopher Campbell (christopher.campbell@univie.ac.at)

Review Timeline:

Submission Date:	22nd Apr 22
Editorial Decision:	24th May 22
Revision Received:	9th Sep 22
Editorial Decision:	18th Oct 22
Revision Received:	8th Nov 22
Accepted:	24th Nov 22

Editor: Hartmut Vodermaier

Transaction Report:

(Note: With the exception of the correction of typographical or spelling errors that could be a source of ambiguity, letters and reports are not edited. Depending on transfer agreements, referee reports obtained elsewhere may or may not be included in this compilation. Referee reports are anonymous unless the Referee chooses to sign their reports.)

1st Editorial Decision 24th May 2022

Thank you for submitting your manuscript on CIN/aneuploidy adaptation to The EMBO Journal. Three expert referees have now assessed it and returned the below-copied reports. As you will see, all reviewers acknowledge the overall quality, presentation and general interest of this work. Nevertheless, they note a number of points that are insufficiently described/discussed and conclusions that would need stronger support. Moreover, referee 1 is not fully convinced that the mechanistic insights derived from the present experiments provide a significant advance over the current understanding of CIN adaptation.

In light of these comments, we would on balance be interested in pursuing a revised manuscript further for publication, in case that you can address the various concerns raised by the reviewers. Among the key issues in this respect would be points 1-3 of referee 3, and points 1 and 4 of referee 1. Furthermore, I feel that the overall impact of the manuscript would be significantly strengthened by attempts to deepen the mechanistic understanding of mutation rescue (ref 1 point 7), and by more directly testing the hypothesis that CIN reduction may be more important than adapting to aneuploidy (ref 1 point 5).

Referee #1:

The manuscript by Clarke et al Multiple routes of adaptation to high levels of CIN and aneuploidy in budding yeast focuses on understanding the long-term adaptation to CIN in budding yeasts. It is a logical continuation of their previous work, where the authors elegantly showed that CIN+ budding yeasts can adapt to the unstable state by acquiring specific karyotypic changes. Here, they describe that the large-scale chromosomal changes are subsequently replaced by targeted mutations in certain genes that reduce the chromosomal instability caused by the BIR1 deletion. This is corroborating the idea emerging during the last 10 years that generally cells under stress first acquire specific aneuploidies that suppress the stress response phenotype, in this case to CIN, and subsequently develop specific point mutations to mitigate the source of the stress; and finally reduce the level of aneuploidy, so that the fitness burden due to cellular response to aneuploidy on the cell does not interfere with efficient proliferation. While this is an interesting and well done. work, it has only a limited novelty. The fact that budding yeast (and other organisms) use large scale karyotypic changes as the first "fast and dirty" response to various stresses has been shown in many different settings (e.g. PMID: 32777450, 26758992, 34555030, 22286062, 23197825 and many more). In fact, aneuploidy is considered the prevalent strategy of fungal adaptation (PMID: 31199875). The authors elegantly showed in their previous paper that this was true even when the stress is caused by CIN+ phenotype, which has been an interesting innovation of that concept. The point mutations obtained here are in predictable processes related to the original CIN causing mutations. Thus, the authors did not show any adaptation to CIN, but rather performed a suppressor screen of bir1 . Their experiments showed that the cells do not adapt to CIN, by rather select mutations that reduce the level of instability.
The authors then go on and characterize the functionality of the specific hypomorphic proteins in the obtained mutated alleles. Not all mechanisms could be clarified, but the authors still make the conclusion that the improved growth is due to suppressing CIN. This is an interesting, thorough work, well written, but it is not clear what is the focus and what is the novelty. The authors try to make conclusions that reducing CIN is more important for improved fitness than adaptation to aneuploidy. This would be an interesting conclusion. They, however, did not rigorously test this. The second key aspect is specifically related to the suppression of bir1 . While this aspect of the manuscript is addressed in more detail and brings more new insights, only some mechanisms could be identified. In conclusion, the manuscript is joining two partly explained stories together. There are several questions which would require clarification.

1. The title should be changed. The authors did not find any adaptation to aneuploidy. They found mutations mitigating this specific type of CIN. They did not show whether another type of CIN would be also reduced by these mutations. They also did not find multiple routes - the obtained mutations, whose mechanism they were able to figure out, work on a similar basis. The main body of the paper is focused on characterization of the MT-kinetochore attachments and their changes. Thus, the title is misleading.
2. The authors state: All mutations listed in Table 1 except for those in MIF2, RTG2, and ASK1 were considered suppressors for this analysis. Why were these genes excluded?
3. Some rescue can be due to altered abundance of specific proteins. The authors did not analyse transcriptome/proteome. It is not necessary to add these experiments, but the authors should mention the possibility. For example, it is not clear whether their sequencing analysis would identify gene amplification.
4. The authors show that the previously identified mutations that increases tolerance of single chromosome gain (ubp6(E256X)) does not improve the growth in their CIN adapted clones. They should try to combine the two mutations - one that reduces high CIN due to the bir1 deletion and one that increases the tolerance of aneuploidy (ubp6(E256X)). Without these experiments they cannot claim conclusions such as (line 420): "Therefore, aneuploidy-tolerating mutations could potentially improve the growth of cells with other sources of CIN that adapt through different aneuploid chromosomes."
5. They work seems to show that the high CIN is a bigger problem than imbalanced karyotype. Would it mean that imbalanced karyotype per se does not bring significant fitness costs in these setting? Their experiments do not exclude it, but also do not bring any new light to this question.
6. While there is generally a decrease of aneuploidy in the strains, and it seems statistically significant, the changes are very small - from 2.9 aneuploid chromosome per cell reduced to 2.6 (Fig. 1F). Do the authors really believe that this makes such a difference to the cells physiologically? It should be stated with caution. The figure 2 F seems much more informative. The authors should consider whether to show the same data in these two different figures. Also, are these means or medians?
7. While the mechanistic analysis of the specific activity of identified mutations is nicely done, there are only limited clear-cut conclusions about the mechanism of action. Also, the testing could be improved. For example, can some of the identified mutation rescue also deletion of SLI15?

Minor:
1. line 233: . . . unattached kinetochores trigger the spindle assembly checkpoint, we first determined if the mutations induce a delay in mitosis . . .". Is this in otherwise wild type, haploid strains? The authors should state this clearly. Also, did they measure the effect of the mutations in the aneuploid cells without the bir1 mutation, but with aneuploid karyotype? While it is not very probable, there is formally the possibility that these mutations do somehow bring also adaptation to aneuploidy, not only to CIN.

Referee #2:

- general summary and opinion about the principal significance of the study, its questions and findings

In the manuscript entitled "Multiple routes of adaptation to high levels of CIN and aneuploidy in budding yeast", Clarke et al. expand on studies reported in 2018 by Ravichandran et al. following the adaptation of yeast cells lacking the CPC component Bir1. These bir1Δ cells were previously found to gain beneficial aneuploidies as they initially adapted to high rates of chromosome missegregation. Now Clarke et al. report further adaptation of these cells after continued clonal expansion. The suppressor mutations reported here represent several distinct and novel classes, which enable adaptation to CPC dysfunction - weakening of kinetochore-microtubule attachments, altering activity of the SAC kinase Mps1, decreasing SCF activity, and altering CPC localization among others. The authors provide good mechanistic experiments showing how suppressors in the Dam1 complex act by weakening kinetochore-microtubule attachments, and this is the primary strength of the manuscript. While the authors do not determine a mechanism for the other classes of bir1Δ suppressors, their observations are still valuable and represent a significant advance in our understanding of how cells adapt to CIN and aneuploidy. This is especially relevant for our understanding of human tumor cell evolution. We therefore recommend this manuscript to be accepted for publication, after the authors address the following minor concerns.

- specific major concerns essential to be addressed to support the conclusions
None

- minor concerns that should be addressed
1. Figure S4C and Lines 344-354. We found following the logic of this section and interpreting the data to be challenging. It would help interpretation if the text and figure were clarified. Clarifications should include, but are not limited to: genotypes compared in the text should be plotted on the same axis, and expanded description provided in the text. What is the phenotype of the mps1-as1 strain alone? Does "WT" refer to WT with respect to MPS1? Why are the doubling times of WT and WT + DMSO so different?

2. Figure 5B, 5C, S3. The authors should describe how to interpret the difference between kinetochore and spindle localized Dad3 better. Line 320-323 "the decrease in localization for the Dam1c mutants was much more pronounced along the anaphase spindle than at anaphase kinetochores, suggesting that the localization phenotypes are more likely to result from defects in microtubule binding than kinetochore association." Are there previous studies that suggest differences in spindle associated and kinetochore proximal are due to microtubule-binding deficient and oligomerization/kinetochore-association, respectively? Also, what exactly was being analyzed, e.g. are the spindle intensity line scans between Nuf2 foci?

3. Figure 6B and lines 374-387. Following the logic of this sections would be easier if the authors propose a loose model for how they believe SCFCdc4 is operating with respect to CPC recruitment. Does SCFCdc4 repress a factor that promotes CPC recruitment possibly? Further discussion of this point either in the Results or Discussion would be helpful.

4. Line 347. Is "improved the doubling time" the most accurate description of the result? Was it increased or decreased instead?

5. The growth assay in Fig 1C-D, Fig 2 D-E (and elsewhere) should be described in more detail. The figure legend states " [strains were] measured by area of colony growth after serial dilution" (line 638). It is unclear from the methods section how this was accomplished. Additional text or providing pictures in supplemental material would help.

6. Using active voice instead of passive voice in Intro and Discussion, including but not limited to line 36, 37, 64-65, 460, would be beneficial.

7. Figure S2B it is surprising that the "WT" strain has a spore viability of 75%. Can the authors please account for this observation, perhaps via a comment in the supplemental figure legend, especially given the "expected" near 100% viability for some of their mutants?

8. Lines 104 and 114. When referring to loss of the gene BIR1 the authors use "Bir1 deletion", which is the convention for referring to proteins. They should instead use "BIR1 deletion" or "bir1Δ"

9. Line 379. There is an unnecessary period after CPC on this line.

10. Line 430 has a typo, MCAK and Kif2b are kinesins not kinases.

- any additional non-essential suggestions for improving the study (which will be at the author's/editor's discretion)
1. The suppressor in the Ndc80 protein that was identified (K181N) appears very similar to a residue mutated in the human Ndc80 by Ciferri et al. in their 2008 study (K146). This K146 was shown to be important for in vitro microtubule binding. Maybe the authors could reference the Ciferri study as it supports their findings.

2. Figure 2C. The cartoon representation of mini-chromosomes as circles is not very helpful. The authors could clarify what these circles are, perhaps including a URA3 marker.

3. Line 267. Could the authors provide more explanation of what the function of Dam1 phospho-mimic mutations are or what they are expected to do based on previous literature.

4. Figure 4C. There is a discrepancy between the results for the Dam1-3D construct in this assay and the other experiments in Figure 4. Could the authors comment on why Dam1-3D might have behaved differently in this assay.

5. Figure 6A. Comment on why do the cdc4-1 strains have such a notable growth defect on -URA?

Referee #3:

In this work, the authors use a panel of S. cerevisiae strains evolved to manage chromosome instability (CIN) that is induced by deletion of the BIR1 gene that is required for proper chromosome segregation. Cells lacking BIR1 display high rates of CIN due to aberrant connections between the microtubules and kinetochores. In a previous publication, these authors evolved strains to adapt to BIR1 deletion; whereas their previous paper studied evolved mutations after shorter-term adaptations, here they

characterize strains that adapt to longer-term bir1Δ. Putting the results together produces a model for the dynamic adaptation to CIN, which has great relevance to cancer evolution since most cancers display both aneuploidy and CIN.

This is an interesting study that adds to past work expanding on evolutionary pathways of adapting to CIN. The direct relevance to cancer is likely to capture the attention of a broad audience. I am not an expert in all the cell cycle details, but I found the paper interesting, well crafted, and overall well controlled. The paper does an excellent job getting into mechanisms and models with what seem to me to be well reasoned hypotheses and experiments.

I had a number of major points that need to be addressed before publication and several other minor points.

1) First, there are insufficient details on the sequencing analysis. The authors have one sentence directing the reader to their 2018 paper for methods, but that is not sufficient since many of the conclusions rely on that analysis; furthermore, even the details in their previous paper are not sufficient. Details on variant calling methods, thresholds for calling variants, and especially how aneuploids versus segmental copy-number changes were determined are required for readers to assess and replicate the results.

2) Equally important, I do not see that the sequencing data (in this publication or their previous publication) is publicly available. This is unacceptable. Sequences should be deposited into repository such as SRA before publication.

3) Second, I had to dig into the supplement to find that this work was done in laboratory strain W303 - by now there have been multiple papers showing that this strain is highly sensitive to aneuploidy and differs from other lab and non-lab strains. The authors need to at least cite the strain background in the main Methods of the paper. I would also like to see at least a mention in the Discussion that the dynamics of CIN adaptation and perhaps even the mechanisms of adaptation could vary in other strains that are inherently more tolerant of CIN.

Other more minor points:

4) The manuscript cites that Sli15 half-life is not altered in cdc4 mutants when cells are treated with cycloheximde (Fig S5), but that Sli15 localization is disrupted (Fig 6). However, it seems that overall abundance of Sli15 should be quantified in the experiment shown in Fig 6 to really lock down that it is localization, and not overall abundance, that is different here. The results shown in Fig S5 are not the same setting and cycloheximide can extend may protein half-lives.

5) Figure 1E compares the # of aneuploid chromosomes per strain, but once again without any methods on how aneuploidy was called this is not convincing or interpretable.

6) Several supplemental figures or panels within would be useful to put into the main paper, including Fig S1A, B, and D. I did not see how "CIN-related genes" were defined for Fig S1. Fig S4C also seemed useful to put in the main document.

7) Some of the figure legends could use clarifications:

7a) In Fig 1C-D and others, the legend describes growth rate but the figure lists relative growth - what is the growth rate normalized to?

7b) Also the legend cites that growth was based on colony size after serial dilution - but is this really single colony size, or density from a patch on a drop assay that is based on serial dilution? Clarity here would help.

7c) Figure 5 should mention what Nuf2 is and why it is relevant (I realize it's listed in another legend, but it should be mentioned here as well.

We would like to thank all of the reviewers for their insightful and constructive comments. We feel that these suggestions have substantially improved the manuscript.

Referee #1:

The manuscript by Clarke et al Multiple routes of adaptation to high levels of CIN and aneuploidy in budding yeast focuses on understanding the long-term adaptation to CIN in budding yeasts. It is a logical continuation of their previous work, where the authors elegantly showed that CIN+ budding yeasts can adapt to the unstable state by acquiring specific karyotypic changes. Here, they describe that the large-scale chromosomal changes are subsequently replaced by targeted mutations in certain genes that reduce the chromosomal instability caused by the BIR1 deletion. This is corroborating the idea emerging during the last 10 years that generally cells under stress first acquire specific aneuploidies that suppress the stress response phenotype, in this case to CIN, and subsequently develop specific point mutations to mitigate the source of the stress; and finally reduce the level of aneuploidy, so that the fitness burden due to cellular response to aneuploidy on the cell does not interfere with efficient proliferation. While this is an interesting and well done. work, it has only a limited novelty. The fact that budding yeast (and other organisms) use large scale karyotypic changes as the first "fast and dirty" response to various stresses has been shown in many different settings (e.g. PMID: 32777450, 26758992, 34555030, 22286062, 23197825 and many more). In fact, aneuploidy is considered the prevalent strategy of fungal adaptation (PMID: 31199875). The authors elegantly showed in their previous paper that this was true even when the stress is caused by CIN+ phenotype, which has been an interesting innovation of that concept. The point mutations obtained here are in predictable processes related to the original CIN causing mutations. Thus, the authors did not show any adaptation to CIN, but rather performed a suppressor screen of bir1Δ. Their experiments showed that the cells do not adapt to CIN, by rather select mutations that reduce the level of instability. The authors then go on and characterize the functionality of the specific hypomorphic proteins in the obtained mutated alleles. Not all mechanisms could be clarified, but the authors still make the conclusion that the improved growth is due to suppressing CIN. This is an interesting, thorough work, well written, but it is not clear what is the focus and what is the novelty. The authors try to make conclusions that reducing CIN is more important for improved fitness than adaptation to aneuploidy. This would be an interesting conclusion. They, however, did not rigorously test this. The second key aspect is specifically related to the suppression of bir1Δ. While this aspect of the manuscript is addressed in more detail and brings more new insights, only some mechanisms could be identified. In conclusion, the manuscript is joining two partly explained stories together. There are several questions which would require clarification.

We agree that the concept of the "fast and dirty" response to stress occurring through aneuploidy is not new. We and many others have observed this for a wide variety of stresses. One of the things that this study adds to the field is what happens as the next evolutionary step after the rapid adaptation through aneuploidy. We demonstrate that the acquisition of point mutations, specifically affecting the perturbed pathway, then alleviates the stress and allows for a return towards euploidy. This connection between the initial adaptation via aneuploidy, the next steps in the adaptation, and the relationship between the two has never been observed before to our knowledge.

We do not believe that all of the identified point mutations were predictable. The SCF complex has not been previously implicated in CPC function and the loss-of-function MPS1 mutations are quite counterintuitive given the known functions of the kinase. Furthermore,

the mutations identified in the Dam1 complex add to our understanding of the function of the complex.

As you will see below, we have attempted to more convincingly show that the CIN reduction is more important than adaptation to aneuploidy through a number of new experiments, many of which were suggested by the reviewer.

By "adapt to CIN", we mean that the cells had high levels of CIN and then they found ways to adapt. The reviewer is correct that the methods of adaptation observed reduced the levels of CIN, and the cells therefore did not adapt to ongoing CIN at the same levels as when the adaptation started. We did not mean to imply that they adapt by becoming less sensitive to continuous CIN and have tried to clarify this point further.

1. The title should be changed. The authors did not find any adaptation to aneuploidy. They found mutations mitigating this specific type of CIN. They did not show whether another type of CIN would be also reduced by these mutations. They also did not find multiple routes - the obtained mutations, whose mechanism they were able to figure out, work on a similar basis. The main body of the paper is focused on characterization of the MT-kinetochore attachments and their changes. Thus, the title is misleading.

Based on this comment, we now understand that the title can be interpreted differently than how we intended. The title was meant to reflect that we started with cells that have very high rates of CIN and aneuploidy and determined how they become more fit over time ("adapted"). We found that different "routes", or pathways were altered to allow the cells to grow better from this initial state of CIN and aneuploidy. Although we did not obtain precise mechanisms for all of the mutations, we characterized them in ways that demonstrate that they work through different mechanisms (Figures 3A-C, EV2B, and EV4D). We have now changed the title to "Adaptation to high rates of CIN and aneuploidy through multiple pathways in budding yeast". Hopefully, this makes our intentions clearer.

2. The authors state: All mutations listed in Table 1 except for those in MIF2, RTG2, and ASK1 were considered suppressors for this analysis. Why were these genes excluded?

Good question and we should have made this clearer in the text. These mutations were excluded because they did not rescue the *BIR1* deletion growth defect when directly tested (Figure 2B). We have now amended the sentence as follows: "All mutations listed in Table 1 were considered suppressors for this analysis, with the exception of genes with mutations that did not rescue the *BIR1* deletion growth phenotype (*MIF2*, *RTG2*, and *ASK1*)." line 208

3. Some rescue can be due to altered abundance of specific proteins. The authors did not analyse transcriptome/proteome. It is not necessary to add these experiments, but the authors should mention the possibility. For example, it is not clear whether their sequencing analysis would identify gene amplification.

It is true that epigenetic or copy number changes could also contribute to the adaptation by altering protein abundance. However, the strong correlation between the identified suppressor mutations and the increased fitness of the strains (Figures 2D and 2E) indicates that the mutations that we identified provide a large majority of the adaptation in these experiments. We have addressed this issue by adding the following sentence: "Other genetic or epigenetic changes that alter protein abundance could also contribute to adaptation under these conditions; however, the strong correlation between the identified suppressor

mutations and the growth phenotype indicate that these alterations would have a relatively small contribution." line 214

We have now added additional details on the sequencing analysis (see additions to the methods section). Our analyses focused on large chromosomal alterations and point mutations, since these things most impact aneuploidy and the observed adaptation respectively. However, we also looked at segmental copy number changes using 4kb bins as now additionally described in the methods.

4. The authors show that the previously identified mutations that increases tolerance of single chromosome gain (ubp6(E256X)) does not improve the growth in their CIN adapted clones. They should try to combine the two mutations - one that reduces high CIN due to the bir1 deletion and one that increases the tolerance of aneuploidy (ubp6(E256X)). Without these experiments they cannot claim conclusions such as (line 420): "Therefore, aneuploidy-tolerating mutations could potentially improve the growth of cells with other sources of CIN that adapt through different aneuploid chromosomes."

As suggested, we combined the Ubp6 mutations with Dam1c, Cdc4, and Mps1 mutations to determine if they act synergistically. The Ubp6 mutations had no affect on the degree of rescue for the other mutations following *BIR1* deletion. These results have been added to the manuscript in **new Figure EV1A**.

5. They work seems to show that the high CIN is a bigger problem than imbalanced karyotype. Would it mean that imbalanced karyotype per se does not bring significant fitness costs in these setting? Their experiments do not exclude it, but also do not bring any new light to this question.

This is an interesting and complex question. There are in essence two types of aneuploidy that are relevant to high levels of CIN. First, there is the largely random missegregation of chromosomes that will create aneuploidy of every chromosome in individual cells in the population. Our population-level sequencing does not detect these types of aneuploidies (many of which will simply result in lethality due to full chromosome loss), so they are inferred from the minichromosome missegregation rates. These aneuploid chromosomes are likely the biggest negative consequence of CIN and provide the selective pressure to decrease CIN in the population as a whole. Second, there is the accumulation of specific aneuploidies within the population that reduce the levels of CIN (for *BIR1* deletion in haploids, these are gains of chromosomes 1, 2, 3, 8, and 10). In a previous publication from our lab, we measured the burden of this second type of karyotype imbalance by adding *BIR1* back to strains than had adapted through acquiring specific aneuploidies (Ravichandran et al. 2018, Figure S1F). We found that there is indeed a cost of these aneuploidies, which is likely why they are decreased in populations that obtain point mutations that decrease the level of CIN (Figure 2F). To determine if the remaining fitness defects in the further adapted strains with CIN-reducing mutations are now more due to the aneuploidy or the remaining CIN, we once again added *BIR1* back in three of the *bir1Δ-ad2* strains (see **new Figure EV1C**). The fitness of the cells was greatly improved by the reintroduction of *BIR1*, indicating that the residual CIN is still the main source of decreased growth in these cells. So, in summary, we conclude that while the addition of extra chromosomes at the whole population level has a deleterious effect on growth (Ravichandran et al. 2018), these defects are not as significant as those caused by CIN. We feel that these results do indeed shed new light on this question.

6. While there is generally a decrease of aneuploidy in the strains, and it seems statistically significant, the changes are very small - from 2.9 aneuploid chromosome per cell reduced to 2.6 (Fig. 1F). Do the authors really believe that this makes such a difference to the cells physiologically? It should be stated with caution. The figure 2 F seems much more informative. The authors should consider whether to show the same data in these two different figures. Also, are these means or medians?

We completely agree that the results from Figure 2F are much more informative than those in Figure 1F, as they now take into account the contributions of suppressor mutations in the adapted populations. Therefore, the minor difference seen in Figure 1F is due to the presence of many strains that did not acquire beneficial point mutations, did not greatly increase in fitness, and did not decrease their aneuploidy burden. Only some of the adapting populations have a strong physiological difference, and the difference between the populations that have or have not adapted substantially are determined in the experiments shown in Figure 2. Hopefully this helps clarify how we interpret those results.

The graph for the figure shows the means and this has now been added to the figure legends. Thank you for pointing out this oversight.

7. While the mechanistic analysis of the specific activity of identified mutations is nicely done, there are only limited clear-cut conclusions about the mechanism of action. Also, the testing could be improved. For example, can some of the identified mutation rescue also deletion of SLI15?

We agree that a full mechanistic characterization of all of the identified mutations is not presented in this study. However, we do come to some interesting mechanistic conclusions.

First, the Dam1 complex likely acts through destabilizing the formation of multimeric rings that promote microtubule interaction (Figures 5, EV2, and EV3). We have added two new experiments related to this point. First, we combined the *duo1(P17L)* mutation with *SLI15* deletion as suggested by the reviewer. We focused on the Dam1c mutant in this experiment since the SCFc and Mps1 mutations did not rescue the ipl1-ts mutation (Figure 3A). The Duo1 mutation was not able to produce viable colonies when combined with *SLI15* deletion, demonstrating that the mutation does not completely bypass the need for the CPC (**new Figure EV2A**). Second, we combined the ipl1-ts mutation with the double *duo1(P17L), dam1(S20D)* mutant. This experiment was used to test if the degree of KT-MT destabilization would scale with the degree of CPC mutant rescue (**new Figure EV2F**). Indeed, the double mutation showed an extremely strong rescue and the growth of the triple mutant was very similar to the Dam1c double mutant at the restrictive temperature. Intriguingly, the ipl1-ts mutant does not rescue the growth defect of the Dam1c mutants, which also provides insight into the mechanistic nature of this genetic interaction.

Second, the SCF complex increases the localization of the CPC to the chromosomes/spindle in prometaphase in yeast and human cells (Figures 6 EV5, and EV6). We have also added additional experiments to strengthen this conclusion. First, to better demonstrate that the SCF does not alter CPC expression levels, we looked at the protein abundance of Sli15 under the same conditions where the microscopy was performed (**new Figure EV5B**). As was observed previously with cyclohexamide treatment, there were no differences in Sli15 expression. Second, we looked at the localization of the CPC in anaphase. Unlike the increased CPC localization observed in prometaphase during chromosome biorientation with

the cdc4(G439S) mutation, there was no increase in CPC fluorescence on the anaphase spindle (**new Figure EV5C**). This demonstrates that the effect of the SCF complex on CPC activity is specific to prometaphase when the CPC is correcting KT-MT misattachments.

We feel that both of these mechanistic insights (along with additional tools in the identified suppressor mutations) will be very helpful to the chromosome segregation field.

Minor:
1. line 233: . . . unattached kinetochores trigger the spindle assembly checkpoint, we first determined if the mutations induce a delay in mitosis . . .". Is this in otherwise wild type, haploid strains? The authors should state this clearly. Also, did they measure the effect of the mutations in the aneuploid cells without the bir1 mutation, but with aneuploid karyotype? While it is not very probable, there is formally the possibility that these mutations do somehow bring also adaptation to aneuploidy, not only to CIN.

The strain used in the experiments is the haploid wild type strain for the W303 background, with genotype MATa; ura3-1; leu2;3-112; his3-11; trp1-1; ade2-1. To make this easier to determine, we have added the figure numbers to the strain list so that the genotypes for each experiment can be looked up directly.

It is true that since the suppressor mutations so clearly affected the CIN phenotype, we did not directly test their affect on aneuploidy. However, we agree that there is no reason that they couldn't do both, so we added suppressor mutations to strains that were engineered to contain aneuploidy of either chromosome 8 or 10 (**new Figure S1C**). In both cases, the suppressor mutations did not affect the growth of the aneuploid strains. We feel that this new data strengthens our conclusions about the function of the suppressor mutations and we appreciate the suggestion.

Referee #2:

- general summary and opinion about the principal significance of the study, its questions and findings

In the manuscript entitled "Multiple routes of adaptation to high levels of CIN and aneuploidy in budding yeast", Clarke et al. expand on studies reported in 2018 by Ravichandran et al. following the adaptation of yeast cells lacking the CPC component Bir1. These bir1Δ cells were previously found to gain beneficial aneuploidies as they initially adapted to high rates of chromosome missegregation. Now Clarke et al. report further adaptation of these cells after continued clonal expansion. The suppressor mutations reported here represent several distinct and novel classes, which enable adaptation to CPC dysfunction - weakening of kinetochore-microtubule attachments, altering activity of the SAC kinase Mps1, decreasing SCF activity, and altering CPC localization among others. The authors provide good mechanistic experiments showing how suppressors in the Dam1 complex act by weakening kinetochore-microtubule attachments, and this is the primary strength of the manuscript. While the authors do not determine a mechanism for the other classes of bir1Δ suppressors, their observations are still valuable and represent a significant advance in our understanding of how cells adapt to CIN and aneuploidy. This is especially relevant for our understanding of human tumor cell evolution. We therefore recommend this manuscript to be accepted for publication, after the authors address the following minor concerns.

- specific major concerns essential to be addressed to support the conclusions

None

- minor concerns that should be addressed

1. Figure S4C and Lines 344-354. We found following the logic of this section and interpreting the data to be challenging. It would help interpretation if the text and figure were clarified. Clarifications should include, but are not limited to: genotypes compared in the text should be plotted on the same axis, and expanded description provided in the text. What is the phenotype of the mps1-as1 strain alone? Does "WT" refer to WT with respect to MPS1? Why are the doubling times of WT and WT + DMSO so different?

The graph in Figure S4C was mislabeled, as it indicated that "WT" contains the *BIR1* deletion. This is not the case, as the doubling time for *bir1Δ* is ~2.5 hours compared to 1.5 hours for wild type. This has now been corrected. The exact strain for the WT has now been added to the figure legend. In addition, we combined the graphs as suggested to make the comparison in the text (*bir1Δ* + DMSO +/- Mps1-as) as clear as possible. Thanks for pointing out this error.

2. Figure 5B, 5C, S3. The authors should describe how to interpret the difference between kinetochore and spindle localized Dad3 better. Line 320-323 "the decrease in localization for the Dam1c mutants was much more pronounced along the anaphase spindle than at anaphase kinetochores, suggesting that the localization phenotypes are more likely to result from defects in microtubule binding than kinetochore association." Are there previous studies that suggest differences in spindle associated and kinetochore proximal are due to microtubule-binding deficient and oligomerization/kinetochore-association, respectively? Also, what exactly was being analyzed, e.g. are the spindle intensity line scans between Nuf2 foci?

The conclusion that spindle localization will be more due to microtubule binding and kinetochore localization is affected by kinetochore binding is a bit of an assumption on our part. We were not able to find any clear correlations between the two biochemical activities and changes to localization along the spindle in the literature. This is why we did not make too strong of a mechanistic conclusion, but instead speculated on a possible reason for the localization differences. We have now reworded the statement to make it clear that this is a speculative explanation.

We have added additional explanations of the fluorescence intensity quantification methods used in both the figure legends and methods section. We used line scans perpendicular to the spindle and measured the area under the curve.

3. Figure 6B and lines 374-387. Following the logic of this sections would be easier if the authors propose a loose model for how they believe SCFCdc4 is operating with respect to CPC recruitment. Does SCFCdc4 repress a factor that promotes CPC recruitment possibly? Further discussion of this point either in the Results or Discussion would be helpful.

We have now added a hypothesis for SCF involvement in CPC recruitment to the discussion with the sentence "One possibility is that the SCF complex targets the degradation of a CPC recruitment factor." on line 498.

4. Line 347. Is "improved the doubling time" the most accurate description of the result? Was it increased or decreased instead?

"Decreased" is indeed clearer than "improved". Fixed.

5. The growth assay in Fig 1C-D, Fig 2 D-E (and elsewhere) should be described in more detail. The figure legend states "[strains were] measured by area of colony growth after serial dilution" (line 638). It is unclear from the methods section how this was accomplished. Additional text or providing pictures in supplemental material would help.

We have now added an additional figure that illustrates the differences in growth seen in the adapted strains (new Figure EV1C). In addition, we have expanded on the explanation of the quantification method in the methods section (line 594).

6. Using active voice instead of passive voice in Intro and Discussion, including but not limited to line 36, 37, 64-65, 460, would be beneficial.

Looking through the manuscript, the amount of passive voice was indeed excessive. Many instances of passive voice are now edited to balance the writing more toward active voice.

7. Figure S2B it is surprising that the "WT" strain has a spore viability of 75%. Can the authors please account for this observation, perhaps via a comment in the supplemental figure legend, especially given the "expected" near 100% viability for some of their mutants?

It is true that overall spore viability was low for some of the dissections that were performed. These have since been repeated, and only dissections where the WT spore viability was high were used for quantification. The average viability of the WT spores is now close to 100% (Figure EV2C).

8. Lines 104 and 114. When referring to loss of the gene BIR1 the authors use "Bir1 deletion", which is the convention for referring to proteins. They should instead use "BIR1 deletion" or "bir1Δ"

Although we did intend to refer to the protein in this case, since gene deletion should eventually result in protein deletion, it is true that gene deletion more accurately describes what was done. We therefore changed the text to refer to the gene instead of the protein. For consistency, we also made this alteration at dozens of other instances throughout the manuscript.

9. Line 379. There is an unnecessary period after CPC on this line.

This period does mark the end of a sentence. However, it may not have been clear because the next sentence starts with the name of a gene mutation and was therefore lowercase. We have changed the sentence to address this issue.

10. Line 430 has a typo, MCAK and Kif2b are kinesins not kinases.

Thanks for pointing out the typo. Fixed.

- any additional non-essential suggestions for improving the study (which will be at the author's/editor's discretion)
1. The suppressor in the Ndc80 protein that was identified (K181N) appears very similar to a residue mutated in the human Ndc80 by Ciferri et al. in their 2008 study (K146). This K146

was shown to be important for in vitro microtubule binding. Maybe the authors could reference the Ciferri study as it supports their findings.

Although these two residues are not in a region that is highly conserved between the two species, they appear to be equivalent. We have therefore added this comparison to the text on line 151. Thank you for the suggestion.

2. Figure 2C. The cartoon representation of mini-chromosomes as circles is not very helpful. The authors could clarify what these circles are, perhaps including a URA3 marker.

Indeed, the text for URA3 in the minichromosome diagram was too small to read. We have now increased the size of the text and added color for emphasis.

3. Line 267. Could the authors provide more explanation of what the function of Dam1 phospho-mimic mutations are or what they are expected to do based on previous literature.

To provide additional context for these mutations, we have added the following text: "The S20D mutation has been demonstrated to reduce microtubule binding in vitro, whereas the 3D mutant results in activation of the spindle assembly checkpoint (Sarangapani et al., 2013; Jin and Wang, 2013)." Line 281

4. Figure 4C. There is a discrepancy between the results for the Dam1-3D construct in this assay and the other experiments in Figure 4. Could the authors comment on why Dam1-3D might have behaved differently in this assay.

It does indeed seem counterintuitive that the 3D mutation causes a mitotic delay and increased checkpoint signaling without resulting in chromosome missegregation. We have now noted that these phenotypes have been reported before, and may indicate a defect in SAC silencing after biorientation is complete.

5. Figure 6A. Comment on why do the cdc4-1 strains have such a notable growth defect on -URA?

Good question. This confused us as well, but we didn't feel it impacted the conclusion of the experiment. We could speculate that the cdc4-1 and met30-6 mutations cause mini-chromosome loss (of the *URA3*-containing plasmid) due to their role in Cse4 (Cenp-A) regulation (Au et al. PLOS Genetics, 2020). To avoid confusion, we have simply removed the -URA plate from the figure and left the YPAD plate to serve as the control.

Referee #3:

In this work, the authors use a panel of S. cerevisiae strains evolved to manage chromosome instability (CIN) that is induced by deletion of the BIR1 gene that is required for proper chromosome segregation. Cells lacking BIR1 display high rates of CIN due to aberrant connections between the microtubules and kinetochores. In a previous publication, these authors evolved strains to adapt to BIR1 deletion; whereas their previous paper studied evolved mutations after shorter-term adaptations, here they characterize strains that adapt to longer-term bir1Δ. Putting the results together produces a model for the dynamic adaptation to CIN, which has great relevance to cancer evolution since most cancers display both aneuploidy and CIN.

This is an interesting study that adds to past work expanding on evolutionary pathways of adapting to CIN. The direct relevance to cancer is likely to capture the attention of a broad audience. I am not an expert in all the cell cycle details, but I found the paper interesting, well crafted, and overall well controlled. The paper does an excellent job getting into mechanisms and models with what seem to me to be well reasoned hypotheses and experiments.

I had a number of major points that need to be addressed before publication and several other minor points.

1) First, there are insufficient details on the sequencing analysis. The authors have one sentence directing the reader to their 2018 paper for methods, but that is not sufficient since many of the conclusions rely on that analysis; furthermore, even the details in their previous paper are not sufficient. Details on variant calling methods, thresholds for calling variants, and especially how aneuploids versus segmental copy-number changes were determined are required for readers to assess and replicate the results.

We have now greatly expanded the section in the Methods on sequencing and data analysis, including providing all of the requested details. In addition, we have now mentioned in the results that 2 of the strains were omitted from the aneuploidy calculations because they contain segmental aneuploidies. Thank you for helping us make our results assessable.

2) Equally important, I do not see that the sequencing data (in this publication or their previous publication) is publicly available. This is unacceptable. Sequences should be deposited into repository such as SRA before publication.

The bam files for the sequencing data have now been deposited on SRA with the accession number PRJNA870080. We have also added a new supplemental table to provide the relevant information for each of the sequencing files (Appendix Table S3). This suggestion to make our sequencing data available is very much appreciated.

3) Second, I had to dig into the supplement to find that this work was done in laboratory strain W303 - by now there have been multiple papers showing that this strain is highly sensitive to aneuploidy and differs from other lab and non-lab strains. The authors need to at least cite the strain background in the main Methods of the paper. I would also like to see at least a mention in the Discussion that the dynamics of CIN adaptation and perhaps even the mechanisms of adaptation could vary in other strains that are inherently more tolerant of CIN.

The strain background is now mentioned in the results, discussion and methods section, including a reference to the potential strain-dependence of the results as suggested. The new text reads: "We note that we used the same strain background as the studies that identified and characterized the role of *UBP6* in tolerating aneuploidy (W303), which has been shown to have a mutation in the *SSD1* gene that makes them more sensitive to aneuploidy (Hose et al., 2020)." These additions can be found on lines 134, 450, and 531 respectively.

Other more minor points:

4) The manuscript cites that Sli15 half-life is not altered in cdc4 mutants when cells are treated with cycloheximde (Fig S5), but that Sli15 localization is disrupted (Fig 6). However, it seems that overall abundance of Sli15 should be quantified in the experiment shown in Fig 6

to really lock down that it is localization, and not overall abundance, that is different here. The results shown in Fig S5 are not the same setting and cycloheximide can extend may protein half-lives.

This is a great point. Although the addition of cycloheximide can help identify smaller changes in degradation rates by removing the complication of continued protein production, the steady-state levels of the protein are more relevant to the function in the cell. We have therefore performed two experiments to test the levels of protein under more physiological conditions. First, we performed a western blot under the same conditions as were used in the experiment in Figure 6B (new Figure EV5B). There is no observable difference in the expression levels and the measured intensities are nearly identical between wild-type and the Cdc4 mutant. Second, we looked at the fluorescence intensity of Sli15 at a later stage in mitosis after biorientation is complete (new Figure EV5C). In anaphase, there is no difference in mNeonGreen Sli15 signal along the spindle, suggesting that the recruitment levels in pre-anaphase are specific to kinetochore/centromere localization. These additional experiments greatly support the conclusion that the primary phenotype of the Cdc4 mutations is in CPC localization during chromosome biorientation.

5) Figure 1E compares the # of aneuploid chromosomes per strain, but once again without any methods on how aneuploidy was called this is not convincing or interpretable.

The threshold that we used for counting aneuploidy was 1.5. Since we sequenced mixed populations of cells, non-integer copy number measurements are potentially representative of real intermediate values. We therefore used the 1.5 cutoff, as we interpret this to mean most of the population contains an extra copy of the chromosome. This information has been added to the methods section.

6) Several supplemental figures or panels within would be useful to put into the main paper, including Fig S1A, B, and D. I did not see how "CIN-related genes" were defined for Fig S1. Fig S4C also seemed useful to put in the main document.

As suggested, the panels S1A and S1B have been moved to the main figure. We appreciate the reviewer's opinion on the importance of these results.

We did not move Figure S4C to the main figures, as Referee #2 indicated that this was one of the more difficult experiments to interpret and the result was largely negative.

A description of how we created our list of CIN-related genes has been added to the methods on line 588.

7) Some of the figure legends could use clarifications:

7a) In Fig 1C-D and others, the legend describes growth rate but the figure lists relative growth - what is the growth rate normalized to?

This is indeed relative growth not growth rate. The growth is normalized to WT growth on dilution series. This has been clarified in the legend. An example image has now been provided in **new Figure EV1C**.

7b) Also the legend cites that growth was based on colony size after serial dilution - but is

this really single colony size, or density from a patch on a drop assay that is based on serial dilution? Clarity here would help.

Colony size was indeed a misrepresentation of the measurement. A section on how the quantification of the dilution series was performed has now been added to the methods on line 594. We hope that this adds clarity.

7c) Figure 5 should mention what Nuf2 is and why it is relevant (I realize it's listed in another legend, but it should be mentioned here as well.

This information has now been added to the Figure 5 and EV3 legends.

1st Revision - Editorial Decision 18th Oct 2022

Thank you for submitting your revised manuscript for our consideration, and please excuse the delay in its reevaluation. Referees 1 and 3 have now assessed the study and your responses once more, and given their positive overall assessment, we shall be happy to accept the study for EMBO Journal publication, as soon as a few remaining suggestions of referee 1 (see below) have been incorporated, and the following editorial points have been addressed.

Referee #1:

The authors have answered my comments thoroughly and thoughtfully. In fact, the revised version, as well as the answers to the comments are very impressive, I enjoyed reading it. I recommend the manuscript to be accepted for publication. I would have only one small suggestion - the authors show that the CIN levels get reduced by the suppressor mutations, and the data is very convincing. Yet, the aneuploidy is not completely abolished in the suppressors (Fig. 2F). Therefore, maybe a more nuanced conclusion and model would be advisable in figure 6D. There, the authors show as the last stage the "Loss of Aneuploidy". It might be more appropriate to state "Aneuploidy reduction" or so. In fact, the figure 2F shows that the suppressor strains carry 1-2 aneuploid chromosomes. Maybe it suggests that the disadvantage due to 1-2 chromosome aberrations does not lead to a strong selection pressure. This more nuanced conclusion - improved cell survival does not require a complete loss of aneuploidy, but the cells necessarily need to maintain low aneuploidy and low CIN - might be even more interesting. And it is for sure closer to the presented data.

Referee #3:

The authors have done a good job responding to my original concerns, including providing a more detailed methods section (which shows a reasonable analysis) and making the data available.

EMBO Press Author Checklist

Corresponding Author Name: Christopher Campbell	
Journal Submitted to: The EMBO Journal	
Manuscript Number: 111500R	

USEFUL LINKS FOR COMPLETING THIS FORM
The EMBO Journal - Author Guidelines
EMBO Reports - Author Guidelines
Molecular Systems Biology - Author Guidelines
EMBO Molecular Medicine - Author Guidelines

Reporting Checklist for Life Science Articles (updated January
This checklist is adapted from Materials Design Analysis Reporting (MDAR) Checklist for Authors. MDAR establishes a minimum set of requirements in transparent reporting in the life sciences (see Statement of Task: 10.31222/osf.io/9sm4x). Please follow the journal's guidelines in preparing your
Please note that a copy of this checklist will be published alongside your article.

Abridged guidelines for figures
1. Data
The data shown in figures should satisfy the following conditions:
- the data were obtained and processed according to the field's best practice and are presented to reflect the results of the experiments in an accurate and unbiased manner.
- ideally, figure panels should include only measurements that are directly comparable to each other and obtained with the same assay.
- plots include clearly labeled error bars for independent experiments and sample sizes. Unless justified, error bars should not be shown for technical
- if n<5, the individual data points from each experiment should be plotted. Any statistical test employed should be justified.
- Source Data should be included to report the data underlying figures according to the guidelines set out in the authorship guidelines on Data

2. Captions
Each figure caption should contain the following information, for each panel where they are relevant:
- a specification of the experimental system investigated (eg cell line, species name).
- the assay(s) and method(s) used to carry out the reported observations and measurements.
- an explicit mention of the biological and chemical entity(ies) that are being measured.
- an explicit mention of the biological and chemical entity(ies) that are altered/varied/perturbed in a controlled manner.
- the exact sample size (n) for each experimental group/condition, given as a number, not a range;
- a description of the sample collection allowing the reader to understand whether the samples represent technical or biological replicates (including how many animals, litters, cultures, etc.).
- a statement of how many times the experiment shown was independently replicated in the laboratory.
- definitions of statistical methods and measures:
  - common tests, such as t-test (please specify whether paired vs. unpaired), simple χ2 tests, Wilcoxon and Mann-Whitney tests, can be unambiguously identified by name only, but more complex techniques should be described in the methods section;
  - are tests one-sided or two-sided?
  - are there adjustments for multiple comparisons?
  - exact statistical test results, e.g., P values = x but not P values < x;
  - definition of 'center values' as median or average;
  - definition of error bars as s.d. or s.e.m.

Please complete ALL of the questions below.
Select "Not Applicable" only when the requested information is not relevant for your study.

Materials

Newly Created Materials	Information included in the manuscript?	In which section is the information available? (Reagents and Tools Table, Materials and Methods, Figures, Data Availability Section)
New materials and reagents need to be available; do any restrictions apply?	Yes	Materials and Methods

Antibodies	Information included in the manuscript?	In which section is the information available? (Reagents and Tools Table, Materials and Methods, Figures, Data Availability Section)
For **antibodies** provide the following information:<br>- Commercial antibodies: RRID (if possible) or supplier name, catalogue number and or/clone number<br>- Non-commercial: RRID or citation	Yes	Materials and Methods, Figures

DNA and RNA sequences	Information included in the manuscript?	In which section is the information available? (Reagents and Tools Table, Materials and Methods, Figures, Data Availability Section)
**Short novel DNA or RNA including primers, probes**: provide the sequences.	Not Applicable	

Cell materials	Information included in the manuscript?	In which section is the information available? (Reagents and Tools Table, Materials and Methods, Figures, Data Availability Section)
**Cell lines:** Provide species information, strain. Provide accession number in repository **OR** supplier name, catalog number, clone number, and/**OR** RRID.	Yes	Appendix Table S1
**Primary cultures:** Provide species, strain, sex of origin, genetic modification status.	Not Applicable	
Report if the cell lines were recently **authenticated** (e.g., by STR profiling) and tested for mycoplasma contamination.	Yes	Materials and Methods

Experimental animals	Information included in the manuscript?	In which section is the information available? (Reagents and Tools Table, Materials and Methods, Figures, Data Availability Section)
**Laboratory animals or Model organisms:** Provide species, strain, sex, age, genetic modification status. Provide accession number in repository **OR** supplier name, catalog number, clone number, **OR** RRID.	Not Applicable	
**Animal observed in or captured from the field:** Provide species, sex, and age where possible**.**	Not Applicable	
Please detail **housing and husbandry conditions**.	Not Applicable	

Plants and microbes	Information included in the manuscript?	In which section is the information available? (Reagents and Tools Table, Materials and Methods, Figures, Data Availability Section)
**Plants:** provide species and strain, ecotype and cultivar where relevant, unique accession number if available, and source (including location for collected wild specimens).	Not Applicable	
**Microbes:** provide species and strain, unique accession number if available, and source.	Yes	Materials and Methods, Appendix Table S1

Human research participants	Information included in the manuscript?	In which section is the information available? (Reagents and Tools Table, Materials and Methods, Figures, Data Availability Section)
If collected and within the bounds of privacy constraints report on age, sex and gender or ethnicity for all study participants.	Not Applicable	

Core facilities	Information included in the manuscript?	In which section is the information available? (Reagents and Tools Table, Materials and Methods, Figures, Data Availability Section)
If your work benefited from core facilities, was their service mentioned in the acknowledgments section?	Yes	Acknowledgements

Design

Study protocol	Information included in the manuscript?	In which section is the information available? (Reagents and Tools Table, Materials and Methods, Figures, Data Availability Section)
If study protocol has been **pre-registered, provide DOI in the manuscript**. For clinical trials, provide the trial registration number **OR** cite DOI.	Not Applicable	
Report the **clinical trial registration number** (at ClinicalTrials.gov or equivalent), where applicable.	Not Applicable	

Laboratory protocol	Information included in the manuscript?	In which section is the information available? (Reagents and Tools Table, Materials and Methods, Figures, Data Availability Section)
Provide DOI OR other citation details if **external detailed step-by-step protocols** are available.	Not Applicable	

Experimental study design and statistics	Information included in the manuscript?	In which section is the information available? (Reagents and Tools Table, Materials and Methods, Figures, Data Availability Section)
Include a statement about **sample size** estimate even if no statistical methods were used.	Yes	Figure legends
Were any steps taken to minimize the effects of subjective bias when allocating animals/samples to treatment (e.g. **randomization procedure**)? If yes, have they been described?	Not Applicable	
Include a statement about **blinding** even if no blinding was done.	Yes	Matrials and Methods
Describe **inclusion/exclusion criteria** if samples or animals were excluded from the analysis. Were the criteria pre-established?<br><br>If sample or data points were omitted from analysis, report if this was due to attrition or intentional exclusion and provide justification.	Yes	Results
For every figure, are **statistical tests** justified as appropriate? Do the data meet the assumptions of the tests (e.g., normal distribution)? Describe any methods used to assess it. Is there an estimate of variation within each group of data? Is the variance similar between the groups that are being statistically compared?	Yes	Figure legends

Sample definition and in-laboratory replication	Information included in the manuscript?	In which section is the information available? (Reagents and Tools Table, Materials and Methods, Figures, Data Availability Section)
In the figure legends: state number of times the experiment was **replicated** in laboratory.	Yes	Figure legends
In the figure legends: define whether data describe **technical or biological replicates**.	Yes	Figure legends

Ethics

Ethics	Information included in the manuscript?	In which section is the information available? (Reagents and Tools Table, Materials and Methods, Figures, Data Availability Section)
Studies involving **human participants**: State details of **authority granting ethics approval** (IRB or equivalent committee(s), provide reference number for approval.	Not Applicable	
Studies involving **human participants**: Include a statement confirming that **informed consent** was obtained from all subjects and that the experiments conformed to the principles set out in the WMA Declaration of Helsinki and the Department of Health and Human Services Belmont Report.	Not Applicable	
Studies involving **human participants:** For publication of **patient photos**, include a statement confirming that consent to publish was obtained.	Not Applicable	
Studies involving experimental **animals**: State details of **authority granting ethics approva**l (IRB or equivalent committee(s), provide reference number for approval. Include a statement of compliance with ethical regulations.	Not Applicable	
Studies involving **specimen and field samples:** State if relevant **permits** obtained, provide details of authority approving study; if none were required, explain why.	Not Applicable	

Dual Use Research of Concern (DURC)	Information included in the manuscript?	In which section is the information available? (Reagents and Tools Table, Materials and Methods, Figures, Data Availability Section)
Could your study fall under dual use research restrictions? Please check biosecurity documents and list of **select agents and toxins** (CDC): https://www.selectagents.gov/sat/list.htm	Not Applicable	
If you used a select agent, is the security level of the lab appropriate and reported in the manuscript?	Not Applicable	
If a study is subject to dual use research of concern regulations, is the name of the **authority granting approval and reference number** for the regulatory approval provided in the manuscript?	Not Applicable	

Reporting

The MDAR framework recommends adoption of discipline-specific guidelines, established and endorsed through community initiatives. Journals have their own policy about requiring specific guidelines and recommendations to complement MDAR.

Adherence to community standards	Information included in the manuscript?	In which section is the information available? (Reagents and Tools Table, Materials and Methods, Figures, Data Availability Section)
State if relevant guidelines or checklists (e.g., **ICMJE, MIBBI, ARRIVE, PRISMA**) have been followed or provided.	Not Applicable	
For **tumor marker prognostic studies,** we recommend that you follow the **REMARK** reporting guidelines (see link list at top right). See author guidelines, under 'Reporting Guidelines'. Please confirm you have followed these guidelines.	Not Applicable	
For **phase II and III randomized controlled trials**, please refer to the **CONSORT** flow diagram (see link list at top right) and submit the CONSORT checklist (see link list at top right) with your submission. See author guidelines, under 'Reporting Guidelines'. Please confirm you have submitted this list.	Not Applicable	

Data Availability

Data availability	Information included in the manuscript?	In which section is the information available? (Reagents and Tools Table, Materials and Methods, Figures, Data Availability Section)
Have **primary datasets** been deposited according to the journal's guidelines (see 'Data Deposition' section) and the respective accession numbers provided in the Data Availability Section?	Yes	Materials and Methods
Were **human clinical and genomic datasets** deposited in a public access-controlled repository in accordance to ethical obligations to the patients and to the applicable consent agreement?	Not Applicable	
Are **computational models** that are central and integral to a study available without restrictions in a machine-readable form? Were the relevant accession numbers or links provided?	Not Applicable	
If publicly available data were reused, provide the respective **data citations in the reference list**.	Not Applicable	
